# How Deep-Sea Wood Falls Sustain Chemosynthetic Life

**DOI:** 10.1371/journal.pone.0053590

**Published:** 2013-01-02

**Authors:** Christina Bienhold, Petra Pop Ristova, Frank Wenzhöfer, Thorsten Dittmar, Antje Boetius

**Affiliations:** 1 HGF-MPG Group for Deep Sea Ecology and Technology, Alfred Wegener Institute for Polar and Marine Research, Bremerhaven, Germany; 2 Max Planck Institute for Marine Microbiology, Bremen, Germany; 3 Max Planck Research Group for Marine Geochemistry, Carl von Ossietzky University, Institute for Chemistry and Biology of the Marine Environment, Oldenburg, Germany; University of Delaware, United States of America

## Abstract

Large organic food falls to the deep sea – such as whale carcasses and wood logs – are known to serve as stepping stones for the dispersal of highly adapted chemosynthetic organisms inhabiting hot vents and cold seeps. Here we investigated the biogeochemical and microbiological processes leading to the development of sulfidic niches by deploying wood colonization experiments at a depth of 1690 m in the Eastern Mediterranean for one year. Wood-boring bivalves of the genus *Xylophaga* played a key role in the degradation of the wood logs, facilitating the development of anoxic zones and anaerobic microbial processes such as sulfate reduction. Fauna and bacteria associated with the wood included types reported from other deep-sea habitats including chemosynthetic ecosystems, confirming the potential role of large organic food falls as biodiversity hot spots and stepping stones for vent and seep communities. Specific bacterial communities developed on and around the wood falls within one year and were distinct from freshly submerged wood and background sediments. These included sulfate-reducing and cellulolytic bacterial taxa, which are likely to play an important role in the utilization of wood by chemosynthetic life and other deep-sea animals.

## Introduction

Most of the deep seafloor receives very little supply of energy and nutrients, leading to extremely oligotrophic conditions in large parts of the ocean [1]. Sunken wood, whale carcasses, kelp and other food falls present locally and temporally restricted inputs of organic material to the deep sea which are quickly localized and exploited by opportunistic fauna (e.g. [2], [3], [4]), and which rapidly develop into hotspots of diversity [5]. Wood falls may be widely distributed at the seafloor and they have been observed in all oceans and at all water depths [6], though they are likely to be more common off the mouths of rivers, around wooded coastlines, and along shipping routes. Locally enhanced degradation processes at these organic falls can lead to reducing conditions and high sulfide concentrations [7], attracting chemoautotrophic bacteria, both free-living and as symbionts of chemosynthetic fauna (e.g. [7], [8]). Observations of shared and related animal taxa at wood falls, whale carcasses, hydrothermal vents and cold seeps have led to the hypothesis that organic falls may present stepping stones in the evolution and dispersal of chemoautotrophic communities in the deep sea, including those taxa constrained to sulfide- and methane-rich niches for their energy supply [9]–[12]. For example, thiotrophic taxa such as the mussels *Bathymodiolus*, *Idas*, and *Thyasira*, the clams *Solemya* and *Acharax*, as well as the siboglinid tubeworm *Sclerolinum* and several vestimentiferan tubeworms have been found to colonize sunken wood [4], [13]. However, how sulfidic habitats develop at wood falls to attract such chemosynthetic taxa is not well understood, because cellulose and lignin degradation is very slow under anoxic conditions [14]. There is also little known about which bacteria colonize deep-sea wood falls or how the deposition of wood affects surrounding benthic communities [15], [16]. Hence, the aim of this study was to contribute to a better understanding of the microbial ecology and biogeochemistry of wood fall ecosystems and their role as biological and biochemical hotspots in the deep sea. Therefore we deployed and revisited four replicate wood falls at different distances to an active cold seep of the Central Nile deep-sea fan (Eastern Mediterranean) [17], [18], to study the colonization of the wood and the development of biogeochemical gradients. The main objectives of this study were to study biogeochemical effects of wood falls including the development of sulfidic environments, and the identity and role of organisms colonizing the wood. Our main findings support the hypotheses 1) that wood-boring bivalves are key to rapid wood degradation, 2) that core bacterial communities develop in sunken wood aiding cellulose degradation and 3) that their production of sulfide attracts chemosynthetic life to the sunken wood.

## Materials and Methods

### Description of wood colonization experiments

**Figure 1:**
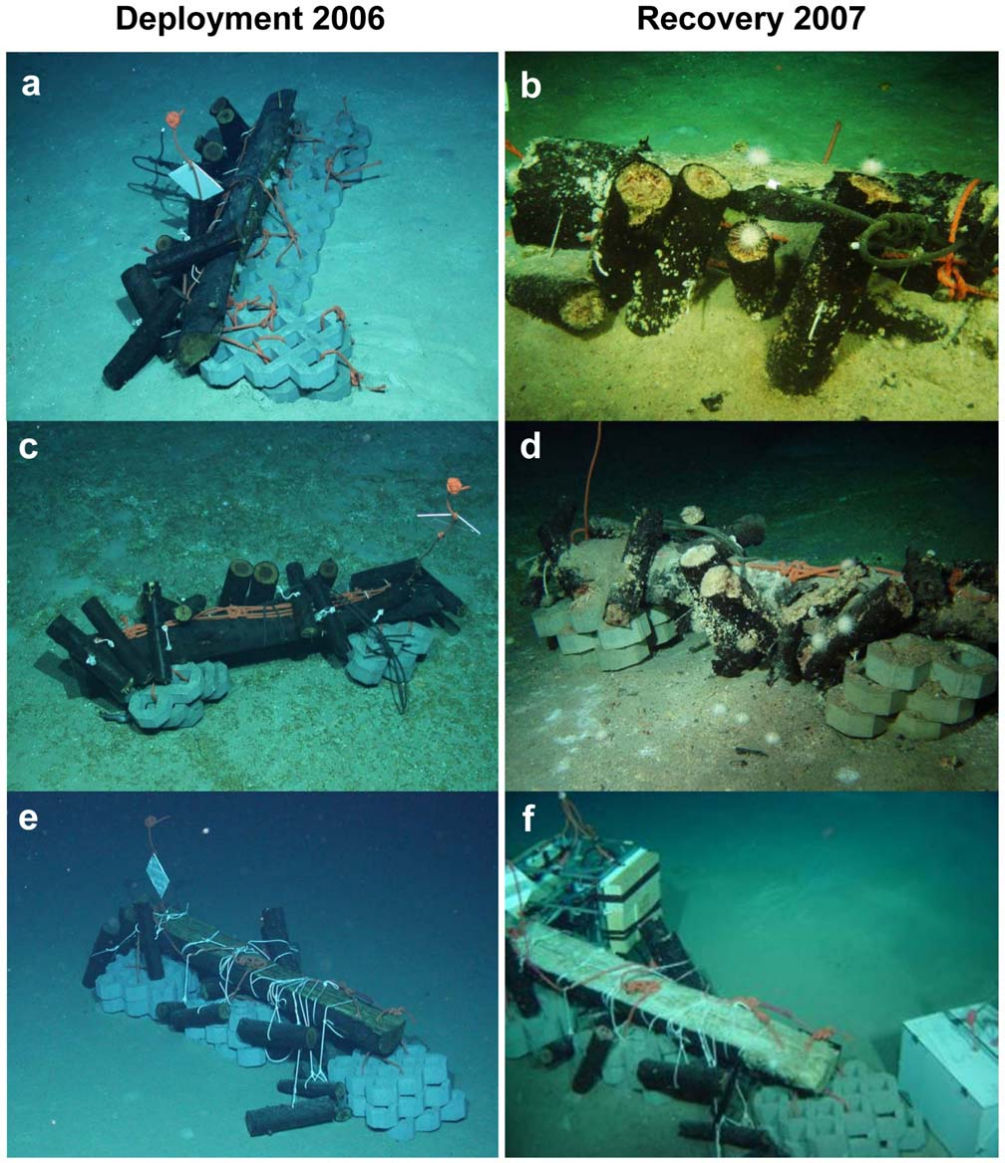
Wood experiments deployed in the Eastern Mediterranean deep sea during the BIONIL cruise in 2006 (RV Meteor) and recovered during the Medeco-2 expedition (RV Pourquoi Pas?) in 2007. a, b) Wood#1 close to carbonate crust, c, d) Wood#2 on carbonate crust, e, f) Wood#5 on sediment. (Pictures a, c, e are courtesy of Marum, University Bremen, Germany; Pictures b, d, f are courtesy of Ifremer, France).

Each of the four replicate wood colonization experiments consisted of one large Douglas fir log (length: 200 cm, diameter: 30 cm) to which several smaller logs (length: 30–50 cm, diameter: 10–15 cm) were attached, as well as cement stones serving as weights (Figure 1). The large logs served as attraction for wood colonizing mega- and macrofauna, while the smaller logs could easily be collected by a remotely operated vehicle (ROV). Three replicate wood parcels (#1, #2, #5) were deployed at the Central Province Site 2A in the Eastern Mediterranean Sea [17], [18] at water depths of about 1690 m during the BIONIL cruise (RV *Meteor M70/2b*) with ROV *Quest 4000* (Marum, Bremen, Germany) in November 2006. A fourth one (#6) was deployed at the same site during the MEDECO-2 cruise (RV *Pourquoi Pas?*) in November 2007 and resampled within less than one day after submersion. Recovery of sub-samples took place with ROV *Victor 6000* (IFREMER, Toulon, France) in November 2007. Characteristic features of the Central Province are pockmark structures (subcircular depressions a few meter in diameter and about one meter deep) associated with active methane seepage and the occurrence of large flat authigenic carbonate crusts above reduced sediments [17]–[20]. Available metadata of both cruises are stored in the PANGAEA database (http://www.pangaea.de/PHP/CruiseReports.php?b=HERMES) and references for the samples are cited accordingly (Table 1, Table S1). Data are deposited under doi:10.1594/PANGAEA.802516.

**Table 1:** Locations of the four wood colonization experiments and PANGAEA references for deployment and recovery of the experiments.

Wood experiment	Location	Position	Date	PANGAEA event label
Wood#1	Close to carbonate crusts	N 32°32.0496 E 30°21.1248	**Deployment**: 19 Nov 2006 **Recovery**: 11 Nov 2007	M70/2b_841_WOOD-1, MEDECO2-D338-PANIER-1
Wood#2	On carbonate crust	N 32°31.9626 E 30°21.1752	**Deployment**: 19 Nov 2006 **Recovery**: 11 Nov 2007	M70/2b_841_WOOD-2, MEDECO2-D338-Wood2-1
Wood#5	On sediments	N 32°32.0790 E 30°21.3840	**Deployment**: 20 Nov 2006 **Recovery**: 13 Nov 2007	M70/2b_846_WOOD-1, MEDECO2-D339-BOX-4, MEDECO2-D339-BOX-5, MEDECO2-D339-BOX-6
Wood#6	Reference, sampled after <1 d at seafloor	N 32°32.0124 E 30°21.1920	**Deployment**: 10 Nov 2007 **Recovery**: 11 Nov 2007	MEDECO2-Wood5-1, MEDECO2-D338-WOOD6-1

**Figure 2:**
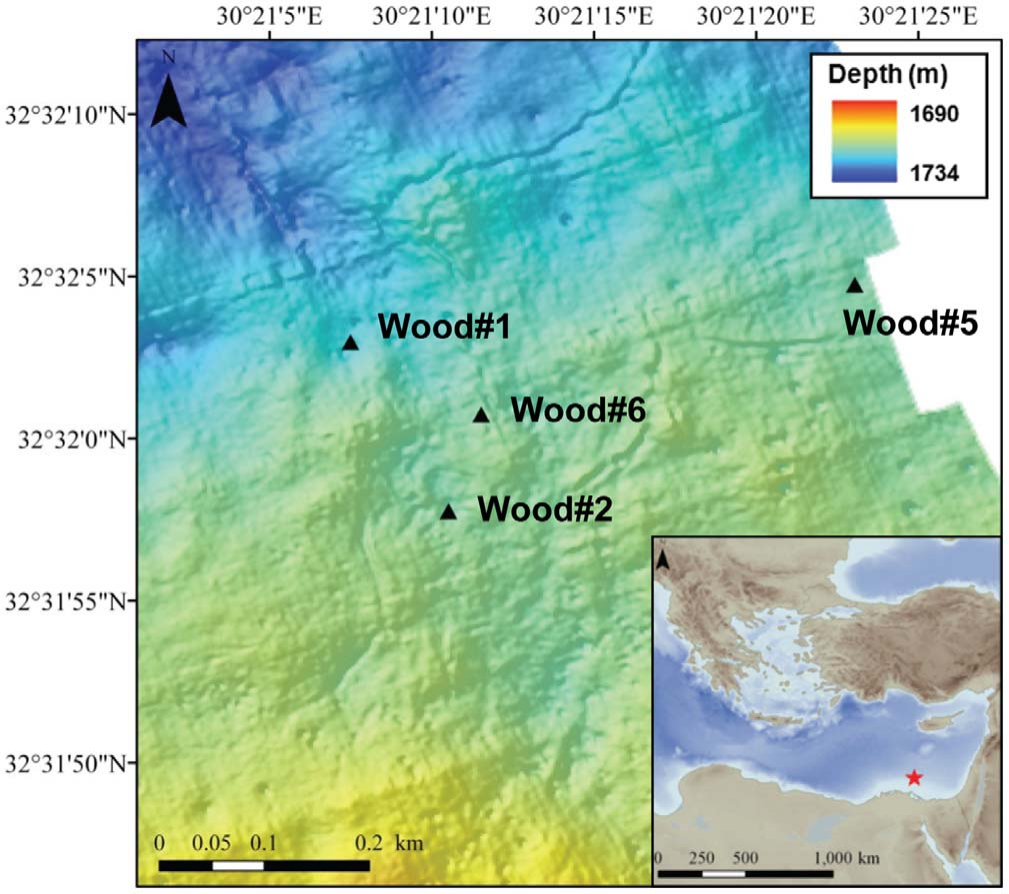
The Pockmark area in the Central Province of the Nile deep-sea fan in the Eastern Mediterranean with locations of the four wood colonization experiments: Wood#1 close to carbonate crust, wood#2 on carbonate crust, wood#5 on sediment, wood#6 close to carbonate crust, sampled after less than 1 day of submersion. The maps were generated in ArcMap (ArcGIS Desktop 9.3) with continental margins provided by ESRI (Kranzberg, Germany) and bathymetry obtained from the 2-minute Gridded Global Relief Data ETOPO2v2 (2006, http://www.ngdc.noaa.gov/mgg/fliers/06mgg01.html). The bathymetry of the Pockmark Area was obtained during Meteor expedition M70/2 (BIONIL) using AUV Aster^x^ equipped with EM120 multibeam (IFREMER/Geosciences Azur).

Wood experiment #2 was deployed on carbonate crust, wood#1 was approximately 2 meters away from carbonate crusts, and wood#5 was located on fully oxygenated pelagic sediments >350 m away from the other two wood experiments, with no indications for past or present gas venting or fluid flow. No methane degassing into the water column was detected at any of the locations. The closest seepage activity, indicated by the observation of bacterial mats [21], was at least 70 m away from any of the wood experiments. Wood experiment #6 was also deployed close to carbonate crusts and served as a reference for freshly submerged wood. Distances between the wood experiments ranged between 31 m between wood#2 and wood#6 and 410 m between wood#1 and wood#5 (Figure 2).

### Visual observation and sampling of wood experiments

To monitor the overall condition of the wood experiments after one year at the seafloor we used high quality video and photo surveys by the ROV. Wood logs were sampled for analyses of the bacterial and macrofaunal wood-colonizing communities, and adjacent sediments were sampled for analyses of wood degradation effects on benthic bacterial communities and biogeochemistry. To avoid loss of organisms, each wood log was put by the ROV manipulator into a separate box closed with a lid. Sampling of the wood logs and handling after retrieval took place at *in situ* temperature of 13°C. From each of the wood experiments wood#1, wood#2, and wood#5, three replicate small logs were collected one year after deployment; for the reference wood#6 one small wood log was collected after less than 1 day of submersion. From each small wood log 2×3 subsamples of the surface (0–2 cm) and the subsurface (2–4 cm) were obtained, cutting 4×4 cm areas on the sides and the middle of the wood log, resulting in 18 subsamples for each wood experiment, and 6 subsamples for wood#6. Any visible organisms (macro- and megafauna) were removed from the wood pieces and wood samples were preserved for DNA extraction (−20°C) and bacterial cell counts (4% Formalin/Seawater). The fauna was collected and preserved for taxonomic analyses. Sediment cores of up to 28 cm length were taken at distances of 0.5 m and 10 m from the wood experiments. Cores were sub-sampled in 1 cm intervals and fixed for DNA extraction (−20°C) and bacterial cell counts (4% Formalin/Seawater). Porewater was extracted for measurements of sulfate, nutrients and dissolved organic carbon (DOC). Additional cores were used for *ex situ* measurements of sulfide, oxygen and pH via microsensors, as well as sulfate reduction (SR) rates. Sediment cores could not be obtained for wood experiment #2 as the carbonate crusts covering the sea floor could not be penetrated with push cores.

Diplomatic permits were obtained for the described field study from the Egyptian authorities (424, 26.04.2009) for sampling in the EEZ (50–200 nm zone). The locations sampled are not privately-owned or protected in any way and the field studies did not involve endangered or protected species.

### Biogeochemical measurements

#### In situ total oxygen uptake (TOU) with benthic chamber

To compare benthic community respiration, *in situ* total oxygen uptake (TOU) was measured at (0.5 m) and away (10 m) from wood#1 using a ROV-operated benthic chamber module [7], [22]. The centrally stirred chamber encloses 284 cm^2^ of sediment with 10–15 cm of overlying bottom water, the latter determined visually with the help of the ROV camera system. At the seafloor, the benthic chamber was operated by the ROV – positioning the chamber at the desired location, gentle insertion of the module into the sediment and starting the measurement. Mounted oxygen electrodes were used to continuously measure the oxygen concentration in the enclosed water body and the TOU flux (mmol m^−2^ d^−1^) was calculated from the initial linear decrease in O_2_ concentration versus time [22], [23].

#### Ex situ microsensor measurements of sulfide, oxygen and pH

Concentration microprofiles of O_2_, H_2_S and pH were determined *ex situ* (laboratory) on retrieved push cores, at (0.5 m) and away (10 m) from wood#1 and wood#5, in order to characterize the establishment of biogeochemical gradients caused by the degradation of wood. Immediately after retrieval, the cores were transferred into an aquarium cooled to *in situ* temperature (13°C). The sediment cores were fully immersed, and the overlying bottom water was gently stirred by an aquarium pump to create a diffusive boundary layer (DBL) thickness close to *in situ* conditions [24], [25]. O_2_, H_2_S and pH microsensors were mounted on a micromanipulator, which allowed measurements of concentration profiles with 1 mm resolution. A Clark-type O_2_ microelectrode, an amperometric H_2_S microelectrode and a pH electrode were used for all *ex situ* measurements [26]–[28]. The sensors were calibrated as described previously [29], [30]. Due to a change of pressure and temperature during ascend the cores could to a certain extent be disturbed when brought on board, thus microsensor measurements were only started once the overlying water in the core was clear. Three replicate profiles of oxygen, sulfide and pH were measured for every investigated core within approximately 12 h of the core retrieval. The results from the H_2_S sensor were converted to total sulfide (ΣH_2_S = H_2_S+HS^−^+S^2−^) concentrations, by using a pK1 for sulfide in seawater after Millero et al. [31].

Fluxes were calculated from the steepest porewater gradients in the sediment, according to Fick's first law of diffusion:
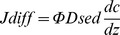
where *J*_diff_  =  diffusive flux [mmol m^−2^ d^−1^], *Φ*  =  porosity, *D*_sed_  =  diffusion coefficient in the sediment [m^2^ s^−1^] and *dc/dz*  =  concentration gradient. *D*_sed_ for oxygen and sulfide is 8.9×10^−10^ and 6.4×10^−10^ m^2^s^−1^, respectively. Sulfide flux was calculated as the sum of the steepest upward and downward fluxes in the sediment.

#### Sulfate concentration

Sediment cores were subsampled in 1 cm depth intervals and transferred into plastic centrifuge vials and centrifuged at 3500×g for 10 min to extract porewater. Subsequently, 500 µl Zincacetate was added to 1 ml porewater and samples were stored at 4°C. Porewater sulfate concentrations were measured in the fixed samples using non-suppressed anion exchange chromatography (Waters IC-Pak anion exchange column, Waters 430 Conductivity detector). As eluent, isophthalic acid (1mmol L21, pH 4.6) containing 10%v/v methanol with a constant flow rate of 1 mL min^−1^ was used. Sulfate concentrations were used for calculations of SR rates.

#### Porosity

Sediments were sampled in 5 cm depth resolution and stored at 4°C. Porosity was determined as the difference in weight of a defined volume of sediment before and after drying at 60°C until constant weight, and data were used for recalculating solid phase wet weight to units of sediment volume in the SR rate calculations.

#### Ex situ measurements of sulfate reduction (SR) and anaerobic oxidation of methane (AOM) rates

Potential rates of bacterial sulfate reduction and anaerobic oxidation of methane in sediments were determined in *ex situ* incubations. Push cores were sub-sampled in triplicate with smaller subcores (diameter: 2.8 cm) immediately after recovery. Radiotracer labelled substrates ^35^SO_4_^2−^ (SR) and ^14^CH_4_ (AOM) were injected in 1 cm intervals following the whole core injection method [32]. Experiments were incubated for 10–12 hours at *in situ* temperature and the reactions were stopped by transferring 1 cm slices of the cores into 20 ml 20% Zincacetate for SR or 25 ml NaOH (2.5% w/v) for AOM. Detailed description of the measurements of methane concentration and radioactivity as well as the calculations of the AOM rates can be found in Treude et al. [33]. Sulfate reduction rates were determined using the cold-chromium distillation method [34]. Calculations of the SR turnover rates were done according to Felden et al. [23].

#### Nutrients

Nitrate, phosphate, silicate, and ammonium were measured from the porewater samples with a Skalar Continous-Flow Analyzer according to the method of Grasshoff et al. [35], to assess whether the deposition and degradation of wood had an influence on the concentration of these nutrients in the environment.

#### Measurements of dissolved organic carbon and total dissolved nitrogen

After extraction of porewater by centrifugation (3500×g, 10 Min), porewater was filtered through 0.22 µm cellulose-acetate filters. Dissolved organic carbon (DOC) and total dissolved nitrogen (TDN) were analyzed with a Shimadzu TOC-VCPH total organic carbon analyzer equipped with a TNM-1 total nitrogen measuring unit. Samples were injected manually (100 µL) in order to minimize the amount of required sample volume [36]. Each sample was injected five times and average values of these injections are reported. Outliers were removed. Before analysis, samples were acidified to pH = 2 with HCl (10 M, p.a.) and purged with synthetic air for 5 minutes to remove inorganic carbon. Detection limits were 5 µM for DOC and TDN (0.06 g C L-1 and 0.07 g N L-1). Analytical errors based on the standard deviations for replicated measurements (at least three measurements per sample) were within 5% for DOC and TDN. Analytical precision and accuracy was tested in each run against deep Atlantic seawater reference material and low carbon water provided by the consensus reference materials program (D.A. Hansell, University of Miami, FL, USA). Procedural blanks, including the filtration step, were obtained with ultrapure water.

DOC flux from the wood-chip covered seafloor accounts for loss of dissolved carbon that has not been incorporated into cell biomass. It was calculated based on Fick's first law of diffusion (see calculation of sulfide and oxygen flux), assuming a porosity of 0.95 for wood chips and 0.65 for sediments. The diffusion coefficient in sediments (D_sed_) was estimated to be 6.2 * 10^−11^ m^2^ s^−1^ [37], [38], based on the assumption that DOC derived from wood is of high-molecular weight.

### Characterization of bacterial communities

#### Bacterial cell numbers

The utilization of wood may support the production of bacterial biomass. Therefore, total bacterial cell numbers in sediment samples were determined using acridine orange direct counts (AODC) based on previously described methods [39], [40]. Single cell numbers were determined for two replicate filters by randomly counting at least 30 grids per filter. The average standard deviation between replicate cell counts was 8% (n = 63). For samples containing wood chips (upper layers next to wood experiments), the duration of sonication (at power: 72/D and a cycle: 30%) was increased from 1 Min 40 sec for sediments to 2×5 Min. Due to the strong fluorescence of wood pieces in acridine orange stained samples, cell numbers for samples containing lots of wood pieces, were verified with cell counts based on the DNA-targeting fluorescent stain 4′,6-diamidino-2-phenylindole (DAPI) [41], which gave much less background fluorescence of wood particles. Pure wood samples (0.3 g) were sonicated in 5 ml 4% Formalin/Seawater (FA/SW) for 3×5 Minutes to detach as many cells as possible without breaking them. After each 5 Minute interval, the FA/SW solution was exchanged after wood pieces had settled for about 3 Minutes. Supernatants from the different sonication steps were combined and volumes for filtering were adjusted to obtain an even distribution of cells on the filters. Additionally, wood pieces remaining after 3×5 minutes of sonication, were again sonicated for 5 minute intervals up to 30 minutes (6×5 Minutes), and the complete volumes filtered, to quantify remaining cells. All samples were kept on ice during sonication. Cell counts for pure wood samples were performed with DAPI staining.

#### DNA extraction

Total community DNA was extracted from 0.3–0.4 g of wood material that had been cut into very small pieces or from 1 g of sediment using UltraClean Soil DNA Isolation Kits (MoBio Laboratories Inc., Carlsbad, CA) and stored in a final volume of 100 µl Tris-EDTA buffer. DNA quantities were spectrophotometrically determined with a NanoDrop ND-1000 Spectrophotometer (NanoDrop Technologies Inc., Wilmington, DE) and adjusted for each step of the molecular protocol.

#### Automated ribosomal intergenic spacer analysis (ARISA)

The fingerprinting method ARISA was used to compare overall bacterial community structure in different samples, as this method allows a high sample throughput. Standardized amounts of 10 ng DNA were amplified in triplicate using bacteria specific ARISA primers ITSF and ITSReub, the latter labeled with the phosphoramidite dye HEX [42]. A standardized amount of PCR product (100 ng DNA) was used for separation of fragments by capillary electrophoresis with the internal size standard MapMarker 1000 ROX (BioVentures Inc., Wahsington DC, USA). Using a standard ARISA protocol, a “fixed window” binning strategy with a bin size of 2 bp was applied to the ARISA generated data to compensate for slight peak shifts between runs and for fragment size calling imprecision [43] (Interactive Binner function, http://www.ecology-research.com). An OTU was considered present if it appeared in one of the three PCR replicates. Relative fluroscence intensities were calculated by dividing each individual peak area by the total area of peaks in a given profile.

#### 454 massively parallel tag sequencing (454 MPTS)

One sample from each wood experiment and surface sediment samples at (0.5 m) and away (10 m) from wood#1 and #5 were selected for 454 MPTS. This allowed for a more detailed description of bacterial communities, because taxonomic assignments can be performed. For cores covered with wood chips (0.5 m), sediment samples were obtained from the previous sediment surface, below the wood chips, for direct comparison with the surface samples from reference cores 10 m away from the wood experiments. Extracted DNA was amplified using primers targeting the V6 region of the bacterial 16S rRNA gene as published on http://vamps.mbl.edu. Fragments were sequenced by pyrosequencing on a Genome Sequencer FLX system (Roche, Basel, Switzerland) at the Marine Biological Laboratory in Woods Hole, MA, USA. Taxonomic assignments were performed with the Global Alignment for Sequence Taxonomy tool (GAST) [44]–[46]. All data were retrieved from the website VAMPS (Visualization and Analyses of Micbrobial Population Structures, http://vamps.mbl.edu). Sequences are deposited in the Genbank Sequence Read Archives (www.ncbi.nlm.nih.gov) under accession number SRA046533.

### Statistical analyses

Overall patterns in bacterial community structure were detected with non-metric multidimensional scaling (NMDS) based on ARISA OTU tables with Bray-Curtis distance as implemented in the R package *vegan* [47]. Analysis of similarity (ANOSIM) was used to assess significant differences between *a posteriori* groupings of samples. All statistical analyses were performed in R (v. 2.9.1) (R Development Core Team 2009, http://www.R-project.org) using *vegan* and custom R scripts.

## Results

### Visual observations of wood experiments

**Figure 3:**
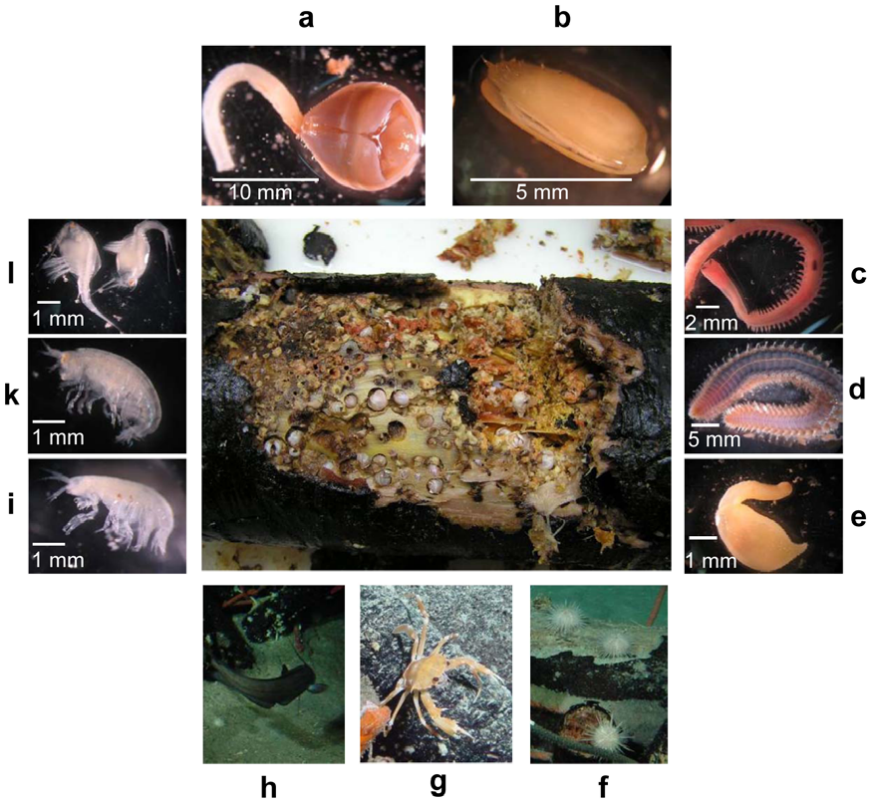
Macrofauna colonizing the wood experiments after one year at the sea floor. a) *Xylophaga dorsalis*, b) *Idas modiolaeformis*, c) *Glycera noelae* sp. nov. d) *Cryptonome* gen. nov. *conclava*, n. sp., e) *Phascolosoma turnerae*, f) *Asterechinus elegans*, g) *Bathynectes piperitus*, h) unidentified deep-sea fish, i, k) unidentified species of amphipods, l) unidentified species of *Leptostracea*. (Pictures h, g, f are courtesy of Ifremer, France (Medeco-2 expedition))

The *in situ* observations after one year of immersion showed that the wood had been heavily degraded by the activity of wood-boring animals (Figures 1 and 3). Abundant burrows and small white shells in the wood indicated the presence of wood-boring bivalves, and a lot of shell debris was observed around the wood logs. The state of degradation and colonization differed between wood logs but also between different positions on one log. Those sides of the wood logs lying in the sediments appeared to be less colonized and degraded by wood-boring animals than those exposed to the bottom waters. In an area of about 0.5–1 m around the wood experiments a layer of fine wood chips produced by wood-boring bivalves had accumulated of up to 5 cm thickness. No distinct bacterial mats were observed on the woods or surrounding sediments, except for one side of wood#2 where a thin mat covered the underlying carbonate. Here, no samples are available because the crusts were too hard for push-coring. Several megafaunal organisms were recorded during *in situ* observations: fish were occasionally observed lingering next to the wood logs, as well as crabs (Portunidae, see below) sitting on wood logs or hiding underneath. In addition, sea urchins (*Asterechinus*, see below) sat on top of wood logs and cement stones (used to weigh down the wood), as well as on surrounding wood chips and sediments. Fauna associated with the wood fall experiments are described in more detail below.

### Faunal diversity at wood falls

#### Wood-boring bivalves

Observations on board confirmed that a strong degradation of the wood logs had occurred during one year at the seafloor for all wood experiments and large numbers of animals had colonized the wood (Figure 3). The degradation of the wood was mainly due to the activity of wood-boring bivalves, identified as *Xylophaga dorsalis*, Turton, 1819 (T. Haga, National Museum of Nature and Science, Tokyo, pers. information); these organisms made up the main biomass of macrofaunal colonizers (Figure 3a). The size of the specimen ranged between 1–10 mm in shell size, and extrapolations of numbers counted in subsections of the small wood pieces, yielded numbers of 100 to >500 individuals per dm^3^. *X. dorsalis* were able to colonize the wood from all sides, especially from the cut ends of the wood logs, but also from the sides and through the bark, indicated by lots of small holes and burrows on the sides of the wood. Even the most inner core (heartwood) showed burrows and individuals of *Xylophaga* in some logs.

#### Chemosynthetic fauna

Small chemosynthetic mussels (*Idas modiolaeformis*) [48] were found on all wood experiments submerged for a year, in, on and directly underneath the bark (Figure 3b). Their shell lengths measured between 1 and 6 mm. Based on subsampling of wood log slices, we extrapolated that up to 150 individuals of *I. modiolaeformis* may have occurred on one small wood log of wood#1 and 30–90 individuals on a small wood log of wood#5 (corresponding to 9–80 individuals per dm^3^).

#### Other fauna

Sea urchins, identified as *Asterechinus elegans* (N. Ameziane, Museum of Natural History, Paris, pers. communication), seemed to be chemically attracted to the wood, as their densities increased towards the wood experiments, e.g., for wood#2 at least 20 sea urchins were counted on and in the close vicinity of the wood (Figure 3f). In addition, crabs (Portunidae; *Bathynectes piperitus* Manning & Holthuis, 1981; B. Richer de Forges, Institut de recherche pour le développement, New Caledonia, pers. communication) were often observed on and under the wood (Figure 3g). Other colonizing macrofauna included a new species of Glyceridae, *Glycera noelae* sp. nov. [49], sipunculids identified as *Phascolosoma turnerae*, Rice 1985 (G.Y. Kawauchi, Department of Organismic and Evolutionary Biology, Harvard University, pers. communication), amphinomids described as a new genus and species of Amphinomidae, *Cryptonome* gen. nov. *conclava*, n. sp. [50], and at least three groups of unidentified species of small crustaceans (Figure 3). There were no qualitative differences in macrofaunal colonization of the three wood experiments that had been submerged for one year. Due to the sampling strategy on board, very small or rare organisms may have not been captured. No macrofauna or megafauna were associated with the control wood#6 after less than 1 day of submergence.

### Biogeochemical characterization

#### Visual observations

**Figure 4:**
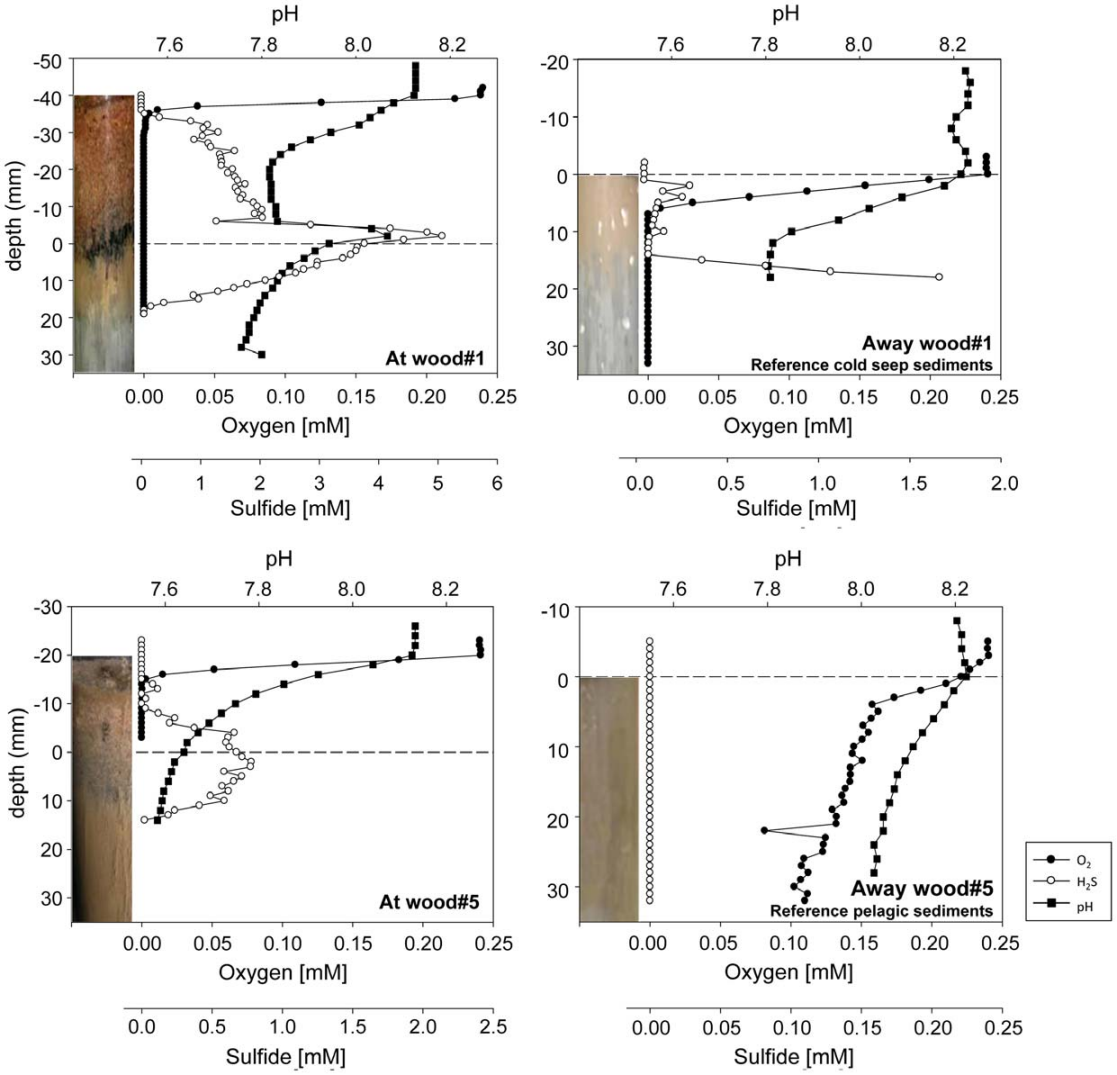
Ex situ microsensor measurements of oxygen, sulfide and pH at (0.5 m) and away (10 m) from wood experiments #1 and #5. Note the different scales for sulfide concentrations.

Around the wood, a thick layer (2–4 cm high) of fine wood chips and fecal matter from wood-boring bivalves covered the seafloor for about 0.5 m around the log (Figure S1). The former sediment surface (0–1 cm depth) below the wood chips was blackened, indicating sulfide production and precipitation with iron as FeS. The subsurface sediments showed a brown to grey color (1–5 cm depth; Figure 4). Control samples from sediment without wood chips (10 m away from wood) differed between experiment#1 and experiment#5: for experiment#1 cores were beige down to max. 4 cm, followed by light gray sediment, changing into dark gray at the bottom 6–8 cm of the cores. For experiment#5, sediments were brown down to 7 cm, then changing into gray/dark gray sediments. These visual observations probably reflect biogeochemical differences in the two regions, with experiment#1 being located close to carbonate crusts, indicating an influence of methane seepage in the past, and experiment#5 being located on pelagic sediments. Several biogeochemical measurements were conducted *in situ* and *ex situ* to describe the influence of the wood on surrounding sediments. The data are available in the PANGAEA database (doi:10.1594/PANGAEA.802516).

#### Sulfide and oxygen fluxes, sulfate reduction and anaerobic oxidation of methane (ex situ) in sediments

The bottom water at the investigated sites had oxygen concentrations of 250 µM (99% saturation at 1700 m water depth and 14°C), and no sulfide was present. Benthic chamber measurements were conducted at (0.5 m) and away (10 m) from wood#1. Total oxygen uptake (TOU) was higher at the wood (25 mmol m^−2^ d^−1^), as opposed to 10 m away (1 mmol m^−2^ d^−1^), evidencing a strongly increased activity of benthic communities influenced by the wood deposition (Table 2).

**Table 2:** Summary of biogeochemical measurements at the wood experiments and at selected seep and reference sites.

	TOU	DOU	OPD	Sulfide flux	SRR
	(mmol m^−2^ d^−1^)	(mmol m^−2^ d^−1^)	(mm)	(mmol m^−2^ d^−1^)	(mmol m^−2^ d^−1^)
At wood#1 (0.5 m, ∼4cm wood chips)	25	4.3±0.9	6.7	31.6±6.7	1.3
Away wood#1 (10 m) (Reference cold seep sediments)	1	2.3±0.4	6.7	15.5±6.1	2.5
					
At wood#5 (0.5 m, ∼ 2 cm wood chips)	n.d.	4.4±0.5	5.0	19.3±7.6	2.0
Away wood#5 (10 m) (Reference pelagic sediments)	n.d.	1.0±0.4	>32	0	0.1^ 1^
Arcobacter mat (Central Province, pockmarks)	71 ^2^	27±16 ^2^	1.75 ^2^	88±64 ^2^	9 – 112 ^2^
Reference	1 ^2^	0.5±0.4 ^2^	>10 ^2^	0 ^2^	0.14±0.04 ^2^

TOU: total oxygen uptake, DOU: diffusive oxygen uptake, OPD: oxygen penetration depth, Sulfide flux: total sulfide flux [H_2_S+HS^−^+S^2−^], SRR: depth integrated sulfate reduction rates over 10 cm sediment depth. *1 Felden, unpubl. data (reference measurement during Bionil cruise 2006, approximately 400 m from wood#5). 2 Grünke et al., 2011.*

At both wood experiments (0.5 m) oxygen penetrated not more than 5–10 mm into the wood chip cover on the seafloor (Figure 4, Table 2). Elevated concentrations of sulfide were detected at both sites within the wood chip layer, approximately coinciding with the black horizon that was observed visually (3–4 cm depth below surface, at the former 0–1 sediment surface layer). Measured total sulfide concentrations at these depths reached up to 5 mM at wood#1 and up to 0.8 mM at wood#5, with calculated maximum sulfide fluxes of 32 and 19 mmol m^−2^ d^−1^ at wood#1 and at wood#5, respectively. Depth-integrated sulfate reduction (SR) rates across 10 cm of the wood – sediment interface were lower (1.3 and 2 mmol m^−2^ d^−1^ at wood#1 and wood#5, respectively). This rather broad range of values may be due to the high heterogeneities in the wood chip layer. No anaerobic oxidation of methane (AOM) was detected at any of the sites, hence sulfide production was related to organoclastic sulfate reduction at all sites. Reference cores at the two sites (10 m away from wood#1 and #5) displayed different profiles. Oxygen penetrated up to 5 mm deep in reference sediments of wood#1, whereas sediments away from wood#5 were fully oxygenated down to at least 32 mm. Diffusive oxygen uptake calculated from oxygen microprofiles was elevated at the wood experiments compared to the reference sites 10 m away (Table 2). Sulfide concentrations reached at least 1.7 mM at about 2 cm sediment depth at the wood#1 reference (close to seep), but no sulfide was present in control sediments away from wood#5.

#### Dissolved organic carbon and nutrient concentrations

The reference pore waters showed dissolved organic carbon (DOC) concentrations around 300 µM. In comparison, the wood chip – sediment boundary layer showed elevated DOC concentrations of 2100 µM at wood#1 and 3000 µM at wood#5. DOC fluxes were 0.4 and 1.3 mmol m^−2^ d^−1^ at wood#1 and wood#5, respectively, and 0 mmol m^−2^ d^−1^ at both reference locations. There was no influence of the wood deposition on phosphate, silicate and nitrate concentrations, but ammonium showed elevated concentrations at the wood chip-sediment boundary layer (3.3–10.6 µM). Ammonium concentrations were similar away from wood#1 (1.9–11.3 µM) and no ammonium was detected away from wood#5.

### Characterization of bacterial communities

#### Bacterial cell numbers

**Figure 5:**
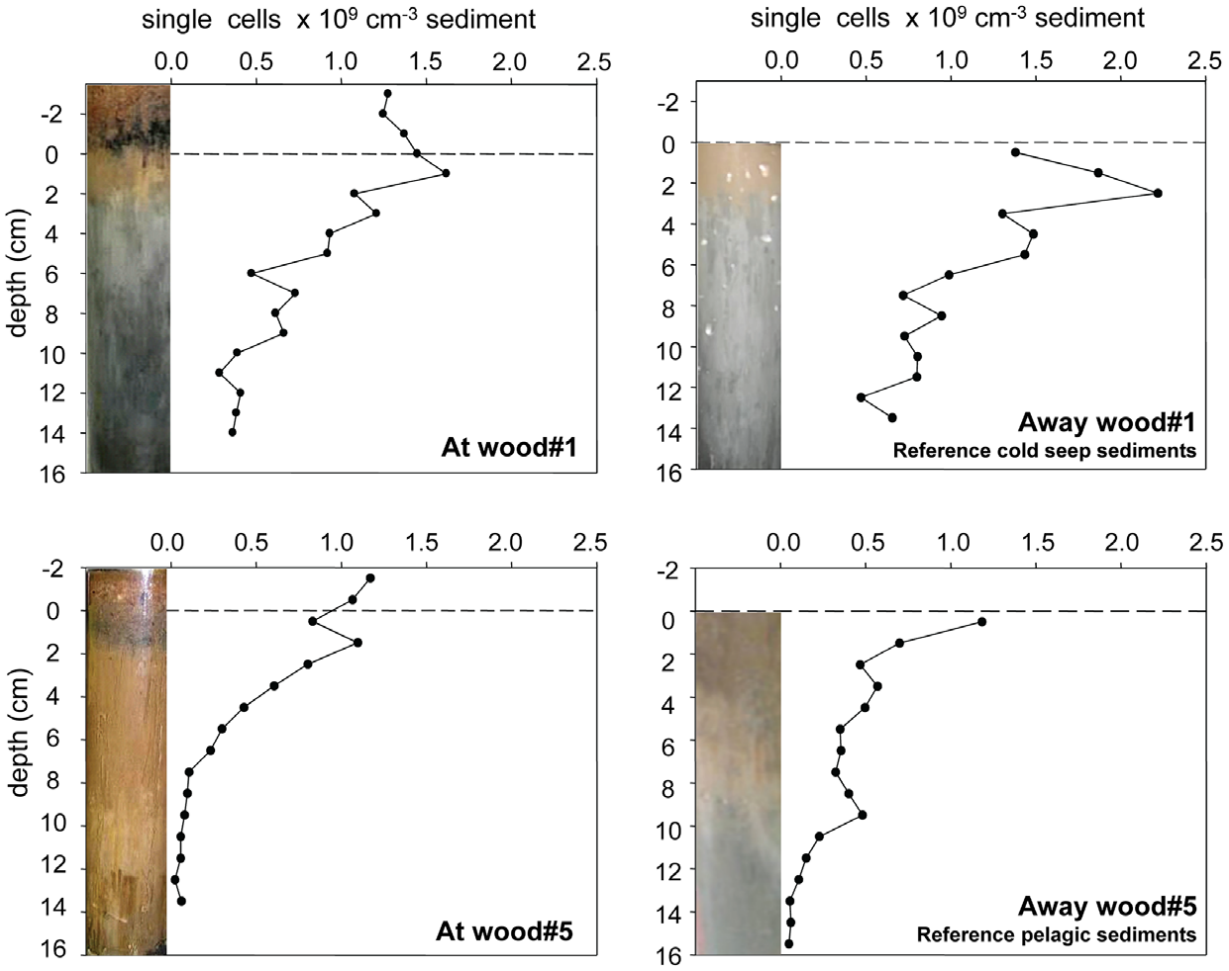
Total bacterial cell numbers at (0.5 m) and away (10m) from wood experiments #1 and #5.

Bacterial cell numbers of pure wood samples ranged between 3.0*10^8^ cells/g wood for wood#2 and #5 and 8.2*10^8^ cells/g wood for wood#1, and were considerably lower for the freshly submerged wood#6 (1.0*10^7^ cells/g wood). The wood chips next to the experiments showed even higher cell numbers with on average 1.2*10^9^ cells/g at wood#1 and 9.3*10^8^ cells/g at wood#5. For wood experiment #1, integrated cell numbers across 10 cm sediment depth (excluding the wood chip layer) showed only small differences between the wood#1 site and its 10 m reference, with 8.9*10^9^ cells/cm^2^ and 1.2*10^10^ cells/cm^2^, respectively (Figure 5). The wood chip layer added 3.7*10^9^ cells/cm^2^. At wood experiment#5 integrated cell numbers across 10 cm sediment depth (3.2*10^9^ cells/cm^2^) were in the range of the reference site (4.7*10^9^ cells/cm^2^). The wood chip layer added 2.8*10^9^ cells/cm^2^. Cell numbers decreased by 70% and 50% from the top cm to 14 cm depth in cores at and away wood#1, respectively, and by 95% in cores at and away from wood#5.

#### Bacterial community structure

**Figure 6:**
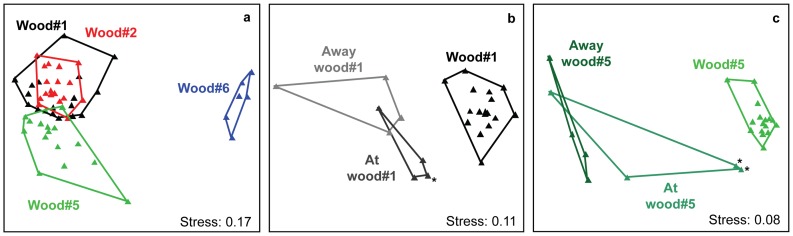
Non-metric multidimensional scaling (NMDS) ordination of relative ARISA OTU abundances with Bray-Curtis distance. Each point represents the consensus of 2–3 replicate ARISA profiles. Colors and groupings indicate the origin of the samples. **a** includes a comparison only of the wood experiments, while **b** and **c** show bacterial community structure on the wood experiments in comparison to surrounding sediments at distances of 0.5 m and 10 m from the wood experiments. Asterisks (*) indicate the presence of wood chips in a sample.

Bacterial community structure determined with ARISA fingerprints showed significant differences between wood and sediment samples (Figure 6, Table S2), but also between individual wood experiments, indicating highly specialized assemblages colonizing the wood falls. The large number of replicate samples enabled us to differentiate between heterogeneity within a single wood experiment and differences between wood experiments from different locations. The most prominent differences were observed between wood experiments that had been submerged for one year when compared to the freshly deployed wood#6, indicating the development of autochthonous communities. Statistically significant differences were also observed between the community structures of the three wood experiments deployed within an area of about 12,000 square meters. No consistent differences were observed for wood samples from the surface or the inner part of the wood samples.

#### Response of specific bacterial taxa to wood input

**Figure 7:**
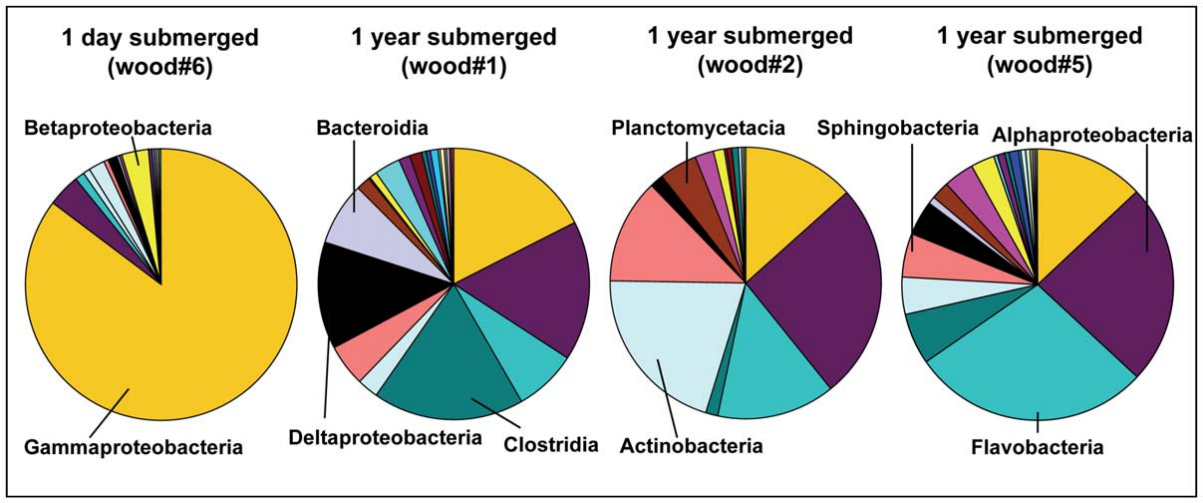
Bacterial community composition of the wood experiments at the class level. Wood#6 served as a control and was sampled after less than 1 day of submergence. Wood experiments #1, #2 and #5 had been submerged for 1 year.

To identify taxa specifically colonizing and responding to the deposition of wood at the seafloor, we applied 454 massively parallel tag sequencing (MPTS) to DNA extracted from woods and sediments, which enables sequencing of samples at a very high resolution as well as taxonomic classification of sequences [44], [45]. Patterns of the relative contributions (relative sequence abundances) of phyla and classes to the overall community clearly differed between the freshly submerged (“control”) wood and the woods submerged for one year, as well as between woods and sediments (Figure 7, Figure S2 and S3). *Proteobacteria*-affiliated sequences dominated in all woods and sediments. The phyla *Actinobacteria*, *Bacteroidetes* and *Firmicutes* showed higher relative sequence abundances in the woods submerged for one year, while the fresh wood#6 was clearly dominated by proteobacterial sequences that were mostly affiliated with *Gammaproteobacteria* (Figure 7). In the woods submerged for one year, *Alphaproteobacteria*, *Flavobacteria*, *Actinobacteria*, and *Clostridia* were present in higher relative abundances. Among the ten most common genera (when only sequences assigned up to genus level were considered) in the freshly submerged wood#6 were *Pseudoalteromonas*, *Vibrio*, *Burkholderia*, *Pseudomonas*, *Erwinia* and *Ralstonia*. In contrast, woods submerged for one year were dominated by sequences affiliated to the genera *Demequina*, *Conchiformibius*, *Blastopirellula*, *Desulforhopalus*, *Thalassobacter*, and *Iamia*. In addition, sequences affiliated to *Teredinibacter* were observed in higher relative abundances in wood submerged for one year while they were absent in control wood#6. None of the ten most common genera were shared between the fresh wood and the woods submerged for 1 year. Wood-chip covered sediments were dominated by *Clostridia*, *Gammaproteobacteria*, *Planctomycetacia* and *Deltaproteobacteria* (Table 3, Figure S2 and S3). The most common genera of the active wood-chip-sediment boundary layer included *Coxiella*, *Ralstonia*, *Methylobacterium*, *Reichenbachiella* and *Desulfobacula*.

**Table 3:** Most common bacterial classes in decreasing order of their relative sequence abundances in wood experiments submerged for 1 day or 1 year, and in wood-influenced or non-wood influenced sediments.

Wood 1 day submerged	Woods 1 year submerged	Sediments at wood experiments (0.5 m)	Reference sediments	Reference sediments
(wood#6)	(wood#1, #2, #5)	(wood-influenced)	(oxygen-limited)	(oxic)
Gammaproteobacteria	Alphaproteobacteria	Clostridia	Gammaproteobacteria	Gammaproteobacteria
Alphaproteobacteria	Flavobacteria	Gammaproteobacteria	Deltaproteobacteria	Acidobacteria
Betaproteobacteria	Gammaproteobacteria	Planctomycetacia	Acidobacteria	Actinobacteria
Actinobacteria	Actinobacteria	Deltaproteobacteria	Holophagae	Alphaproteobacteria
Deltaproteobacteria	Clostridia	Alphaproteobacteria	Epsilonproteobacteria	Holophagae
Flavobacteria	Sphingobacteria	Actinobacteria	Actinobacteria	Deltaproteobacteria
Clostridia	Deltaproteobacteria	Acidobacteria	Clostridia	Planctomycetacia
Sphingobacteria	Bacteroidia	Betaproteobacteria	Betaproteobacteria	Betaproteobacteria
Acidobacteria	Planctomycetacia	Holophagae	Acidobacteria	Gemmatimonadetes
Bacteroidia	Verrucomicrobia	Sphingobacteria	Alphaproteobacteria	Bacilli

As to the comparison between wood-influenced sites and reference sediments, the latter showed a high proportion of *Acidobacteria and Actinobacteria*. *Bacteroidetes* and *Firmicutes* were present with higher relative sequence abundances at and on the woods. *Planctomycetacia* and *Clostridia* showed higher relative sequence abundances in sediments at wood#1, when compared to sediments at wood#5, where *Deltaproteobacteria*, *Sphingobacteria* and *Alphaproteobacteria* showed higher relative sequence abundances. The reference sediments showed differences in composition between the oxygen-limited location 10 m away from wood#1 (*Deltaproteobacteria*, *Epsilonproteobacteria*, and *Clostridia)* and the oxic sediments 10 m away from wood#5 (*Actinobacteria*, *Acidobacteria*, and *Alphaproteobacteria)* (Table 3, Figure S2 and S3).

#### Shared bacterial types (wood-specific types)

A more in-depth analysis of shared bacterial types between samples was performed at the level of operational taxonomic units defined at 3% sequence difference (OTU_0.03_), to avoid masking patterns when pooling sequences into broader taxonomic categories. Singletons, i.e., sequences occurring only once in the whole dataset (36% of the total number of OTU_0.03_) were removed, and shared and unique OTU_0.03_ between samples were calculated with 1000 sequence re-samplings in each sample based on the smallest dataset, to account for differences in sequence numbers between samples. 18% of OTU_0.03_ were shared between the three woods submerged for one year (Figure S4a), containing the majority of all sequences (72%). In addition a considerable proportion of OTU_0.03_ were unique to one sample (14–21%), but in total they only represented 6% of all sequences. In contrast, only 10% of the OTU_0.03_ were mutually shared between control wood#6 (submerged for 1 day) and the three woods submerged for one year (Figure S4b). For pairwise comparisons between woods and their surrounding sediments (0.5 m and 10 m) the largest proportion of shared OTU_0.03_ occurred between wood#5 and the sediment wood-chip boundary layer at wood#5 (23%; other comparisons: 4–5%).

The 30 most sequence abundant OTU_0.03_ that were shared between the three 1-year submerged woods were affiliated with: Actinobacteria (*Iamia, Demequina*), Alphaproteobacteria (*Rhodospirillaceae, Rhodobacteraceae, Hyphomonadaceae, Phyllobacteriaceaea*), Bacteroidetes (*Flavobacteriaceae, Chitinophagaceae, Marinilabiaceae*), Betaproteobacteria (*Neisseriaceae*), Deltaproteobacteria (*Desulfobulbaceae*), Firmicutes (*Lachnospiraceae, Ruminococcaceae, Peptostreptococcaceae*), Gammaproteobacteria (*Thiotrichaceae, Vibrionaceae*), Verrucomicrobia (*Verrucomicrobiaceae*) (Table S4). Of those only two OTU_0.03_, affiliated with *Bacteroidetes* (*Flavobacteriales*) and *Firmicutes* (*Clostridiales*), were shared with control wood#6 (Table S3, S4, S5, S6, S7, S8, S9). Furthermore, none of the 30 most abundant OTU_0.03_ were shared between the wood experiments (1 year submerged) and control sediments (10 m away from wood experiments).

## Discussion

### Evolution of sulfidic niches at deep-sea wood falls

**Figure 8:**
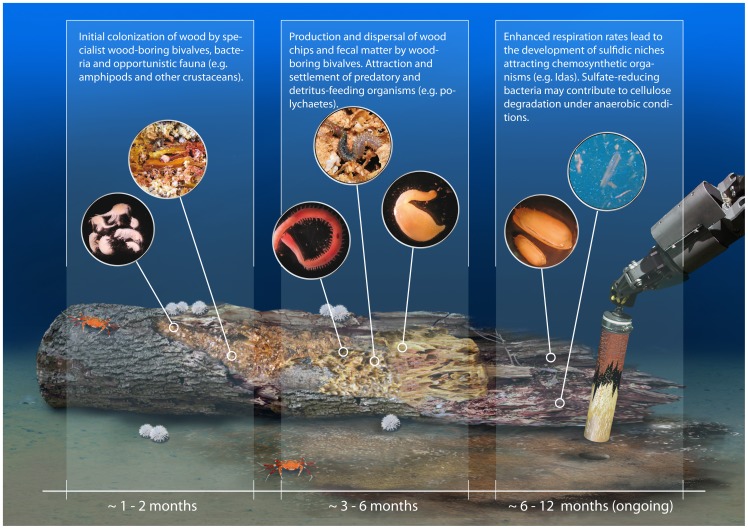
Schematic illustration of the proposed succession of a wood fall during the first year at the deep-sea floor. Organisms shown on inlets from left to right: amphipods and one other type of unidentified crustacean, wood-boring bivalve *Xylophaga dorsalis*, polychaete *Glycera noelae* sp. nov., polychaete *Cryptonome* gen. nov. *conclava*, n. sp., sipunculid *Phascolosoma turnerae*, chemosynthetic bivalve *Idas modiolaeformis*, DAPI-stained bacteria with pieces of wood (Illustration: Sabine Lüdeling, Medieningenieure).

Wood logs that sink beyond the photosynthetic zone of the ocean provide large amounts of organic matter to the oligotrophic deep sea. However, very few organisms can directly degrade wood and use it as an energy and carbon source, because the degradation of cellulose and lignin requires specific digestive enzymes. Furthermore, the degradation of cellulose is generally absent or slow under anoxic conditions [14], [51], [52]. Accordingly, it is known that sunken ships preserve well in cold, salty, anoxic environments [53]. The experiments in the Eastern Mediterranean deep sea showed that wood can be quickly localized and colonized by deep-sea fauna as well as by bacterial communities within a few months of deposition (Figure 8) [2], [54], [55]. Wood-boring bivalves play a key role in the initial degradation of the wood, the dispersal of wood chips and fecal matter around the wood log, and the provision of colonization surfaces to other organisms. In our experiments, wood-boring bivalves of the species *Xylophaga dorsalis* had fully colonized the wood in large numbers after one year (Figure 3a). They were responsible for the rapid degradation and littering of wood chips and fecal matter around the wood log, which lead to enhanced respiration rates and the emergence of sulfidic zones (Figure 8). In addition, *X. dorsalis* provided colonization surfaces for other organisms that were attracted to the wood, such as polychaetes and sipunculids. Wood-boring bivalves may therefore be considered a keystone species in wood fall habitats, as they transform the energy stored in the wood into nutrients that can be digested by other animals, either as feed for predators and scavengers or by their fecal pellets which are used by detritus-feeders [2], [56].

Elevated diffusive and total oxygen uptake rates measured around the wood falls indicated an increased respiratory activity of the entire benthic community due to the degradation of organic matter derived from the wood falls. The development of anoxic zones subsequently enabled sulfide production by sulfate reducing bacteria, and enhanced sulfide fluxes around the wood experiments (Table 2). This specific process associated with wood falls could best be observed at wood#5 which was located on pelagic sediments, where oxygen penetrated several centimeters into the sediment and no sulfide was present at the reference location (Figure 4). Wood#1 in contrast was located close to an active seep site above already reduced sediments. Here sulfide was present also in control samples 10 m away from wood#1. Nevertheless, the addition of wood changed the sedimentary environment in that the sulfide maximum shifted from its subsurface position at 2 cm sediment depth (reference site) to the surface sediment when buried under wood chips. Interestingly, the strongly elevated concentrations of dissolved organic carbon at the wood-chip sediment interface indicated that cellulose degradation was highest under anoxic conditions and hence enabled by anaerobic benthic bacteria, e.g. fermenters and sulfate reducers. This was confirmed by measurements of sulfate reduction, which also peaked at the wood chip-sediment contact zone. These observations demonstrate that, after one year, the presence of wood at the seafloor had led to the creation of sulfidic niches, comparable to what has been observed at whale falls, albeit at lower rates [7], [57].

The higher total oxygen uptake at the woods indicated an increased activity of sedimentary communities around the wood falls. In comparison to other active seep sites in the area, these values were in the range of cold seeps at the Amon mud volcano [58], or in the studied Pockmark area [21] (Table 2). Total oxygen uptake rates at the wood experiments were also similar to values reported for a whale fall in the Santa Cruz Basin, California that had been submerged for 6–7 years [7]. Sulfide concentrations at the wood experiments were elevated, and in the range of or slightly lower than values reported from below a bacterial mat located directly at a whale fall (1–4 mM) [7]. In contrast, waters surrounding hydrothermal vents usually show lower sulfide concentrations (100–750 µM) [59]–[61]. Likewise, sulfide fluxes were higher at the wood experiments when compared to reference measurements (Table 2) [21]. Sulfide fluxes and sulfate reduction rates reported from other chemosynthetic habitats at cold seeps vary widely, and the rates measured here would fall into the lower range of sulfate reduction rates previously observed [21], [23], [62], [63]. Hence, our measurements demonstrate that over a period of one year, wood falls in the deep sea can provide sulfide fluxes similar to those of other chemosynthetic habitats, attracting thiotrophic chemosynthetic symbioses. Accordingly, small chemosynthetic mussels of the genus *Idas modiolaeformis* were mainly found on or underneath the bark of wood pieces from all three experiments (Figure 3b).

### Persistence of sunken wood ecosystems

We used oxygen uptake rates and DOC fluxes around the wood experiments, as well as assessments of bacterial and *Xylophaga* biomass on the wood to estimate the time for a complete mineralization of wood-derived carbon. The total volume of wood deposited on the seafloor was approximately 120,000 cm^3^. With a dry-wood density of 0.51 g/cm^3^ for Douglas fir [64], [65], and a carbon content of 0.5, this results in a total carbon input of ca. 30 kg C for the whole wood experiment. Wood chips covered the seafloor in a radius of 0.5–1 m (average: 0.75 m) around the wood log, making up an area of approximately 5.5 m^2^, to which oxygen uptake rates and DOC fluxes were extrapolated. Carbon mineralization rates estimated from total oxygen uptake rates across the wood-chip covered seafloor (25 mmol m^−2^ d^−1^ at wood#1; Table 2) resulted in a turnover of 600 g C yr^−1^. DOC fluxes were 0.38 and 1.31 mmol m^−2^ d^−1^ for wood#1 and wood#5, respectively, corresponding to 11 and 33 g C yr^−1^ across the wood-chip covered seafloor (5.5 m^2^). Furthermore, carbon incorporated into biomass was estimated based on bacterial cell numbers and *Xylophaga* abundances in the wood. For bacteria an average cell volume of 0.07 µm^−3^ [40] and a biomass conversion factor of 3*10^−13^ g C µm^−3^ biovolume were assumed [66], leading to an estimation of 0.6 g C yr^−1^ converted into net bacterial biomass. *Xylophaga* were measured and their wet weight, dry weight, as well as ash-free dry weight were determined, in order to estimate carbon biomass. Here, rough extrapolations of total abundance estimates and size-frequency distributions of *Xylophaga* in different wood logs led to estimates of 60 to 427 g C yr^−1^ that were incorporated into net animal biomass. Considering the turnover of carbon through respiration and DOC efflux across the wood-chip covered seafloor, as well as carbon incorporated into bacterial and *Xylophaga* biomass, it would take in the order of 35 years until all wood-derived carbon is gone. In comparison, the life-time of whale falls and vents is estimated with decades to centuries [3], whereas the life time of cold seeps can be much longer [67].

### Colonization of sunken woods by deep-sea fauna and bacteria

#### Faunal colonizers

In general, the specific types of colonizers and their succession on different wood falls may depend on a variety of factors, including the geographic location, season, and the type and size of wood. The rapid colonization of the wood experiments in the Nile deepsea fan suggests that terrestrial woody material may have been a regular resource in this oligotorophic environment for a long time, e.g. through input by the Nile river. However, it remains unclear how organisms localize wood falls in the vast deep-sea environment (e.g., chemical cues like the presence of organic matter, degradation products) and what their reproductive and dispersal strategies are. High DOC fluxes at the wood experiments suggest that motile organisms may be attracted by dissolved organic signal molecules.

Wood-boring bivalves depend on wood to maintain their populations and hence must be able to quickly localize and exploit the stochastic falls of wood widely scattered on the ocean floor [2]. The genus *Xylophaga* (family *Pholadidae*) is known to include opportunistic species that colonize wood at depths greater than 150 m [68]. Tyler and colleagues [54] have described the settlement, growth and population dynamics of *X. depalmai*, but it remains unclear how *Xylophaga* larvae and/or adults detect the presence of wood in the deep sea. They are the deep-water counterparts of bivalves of the family *Teredinidae* (also known as “shipworms”) that colonize and ingest wood in shallow waters. *Teredinidae* and *Xylophaga* both have endosymbiotic bacteria in specialized cells located in the gills [68], [69]. For shallow water *Teredinidae*, symbionts have been characterized as cellulolytic and nitrogen-fixing *Gammaproteobacteria* [70], [71] but little is known about the deep-sea *Xylophaga* and their symbionts. Our 454 MPTS dataset revealed sequences affiliated to the 16S rRNA gene sequence of *Teredinibacter turnerae* that has been isolated from the gills of teredinid species and that has been cultured and described as a cellulolytic nitrogen fixing gammaproteobacterium [71]. These sequences occurred in higher relative abundances in wood submerged for one year while they were absent in the fresh wood#6 and in sediment samples away from the wood experiments. *Teredinibacter turnerae* lives as an intracellular endosymbiont in the gills of wood-boring bivalves of the family *Teredinidae* [72]. It possesses cellulases and nitrogenase to convert the cellulose in the wood into digestible carbon and supplement the nitrogen-deficient wood-diet of its host. An initial phylogenetic characterization based on 16 S rRNA gene clone libraries of bacterial symbionts associated with *Xylophaga* from this study, showed that a majority of clone sequences clustered with those of *Teredinidae* symbionts (C. Borowski, pers. communication), suggesting a close phylogenetic relationship between symbionts of the shallow and deep-sea wood-boring bivalves. It is not clear however whether sequences obtained by 454 MPTS analysis originated from free-living bacteria in the wood or from bacteria associated with *Xylophaga* or other macrofauna that may have been crushed during sampling.

The chemosynthetic bivalves colonizing the wood experiments were identified as *Idas modiolaeformis*, which have also been recovered from carbonate crusts at active seeps as well as from other wood colonization experiments in the same study area [4], [48], [73]. The genus *Idas* is known to harbour a variety of bacterial symbionts including sulfide oxidizers [73], [74]; these mussels may therefore serve as indicators of sulfidic conditions in their environment. The higher number of *Idas* individuals found on wood#1 (located close to carbonate structures) may indicate that mussels colonize the wood from carbonate crusts in the Pockmark area, but this remains speculative due to the limited number of experiments. Members of the genus *Idas* have also been found on wood falls in other oceanic regions [55], [75], [76], as well as in association with whale carcasses [5], [57], and hydrothermal vents [77], and therefore seem to be cosmopolitan among chemosynthetic ecosystems in the deep sea.

Sea urchins (*Asterechinus elegans*) were also attracted to the wood (Figure 3f) and had been reported from wood falls in the West-Pacific region [78]. Earlier observations of their gut content have indicated that they are a wood-feeding species and that they might have a microbial community associated with their gut content that would be able to support a digestion of wood [78]. This is supported by the observation of wood particles associated with the gut content of specimen observed in this study.

While diverse gastropoda, polychaeta and bivalvia dominate macrofauna in reduced sediments and carbonate crusts in the Pockmark area [79], the wood falls were only colonized by a very specific set of macrofaunal organisms. Only few amphinomids (polychaeta) have yet been recorded from the deep sea [80], [81], but they have been found at hydrothermal vents [82], [83] and seeps [84], in oxygen minimum zones [85], and in other wood colonization experiments in the Pockmark area [4]. Amphinomids recovered within the framework of this study have been described as a new species and genus (*Cryptonome* gen. nov. *conclava*, n. sp.; (Figure 3d)) [50]. Glyceridae (this study: *Glycera noelae* sp. nov.; Figure 3c) have been reported from other wood colonization experiments [4], as well as from reduced sediments in the Pockmark area [79]. The sipunculid species *Phascolosoma turnerae* (Figure 3e) was originally described as a deep-water species found in association with submerged wood, occupying burrows in the wood [86]. Members of this genus have been reported from earlier colonization experiments in the study area [4], as well as from seeps [87] and whale carcasses [3], [88]. It is not clear, whether the sipunculans enter *Xylophaga* burrows or whether they are able to produce their own burrows [86], but sipunculans have been described to bore into other structures like calcareous rock [89], or to use empty foraminifera tests as shelter [90]. No filter feeders such as crinoids, anthozoa or sponges were observed on the wood falls, they were also absent from the close by carbonate-cemented seafloor. Apparently, the particle-poor, oligotrophic deep-sea bottom waters do not sustain filter feeders in this basin.

While *Xylophaga* are likely endemic to wood falls in the deeper ocean, several of the macrofaunal organisms recovered from our experiments and listed above are shared with whale falls, seeps and/or hydrothermal vents, indicating that these ecosystems may share a close evolutionary history for part of their faunal component [3]. Wood falls present specific hotspots of diversity in the deep sea, attracting a variety of fauna utilizing the wood for different purposes (nutrition, shelter, grazing) [55].

### Bacterial communities

A main objective of this study was to investigate the colonization of sunken woods by bacteria, and to test the hypothesis that core bacterial communities may develop in sunken woods. The degradation of submerged wood as a source of energy and carbon requires complex enzymatic transformation of the macromolecular matter by adapted microbial communities [52], [91]. In the context of cellulose degradation for biofuels, the potential discovery of novel bacterial types and enzymes adapted to high salinity may be of interest for industrial applications [92]. On land, fungi play an important role in the degradation of woody material, but little is yet known about key aquatic microbial organisms responsible for the degradation of cellulose [93], despite it being the second most abundant carbohydrate in the sea and the most abundant on land. Previous studies on microbial communities colonizing sunken woods indicate that bacteria dominate sunken woods compared to fungi or archaea [15], [16], [94], but the main types forming the core community remained unknown.

In the present experiments, wood degradation appeared to be substantially aided by the activities of wood-boring *Xylophaga*, dispersing the wood logs into fine chips and fecal matter, offering a large surface for further microbial degradation. Accordingly, we observed an increase in bacterial cell numbers associated with wood by two orders of magnitude within a year, from the freshly submerged wood with low numbers of 1.0*10^7^ cells/g to the wood chip layer with 9.3*10^8^–1.2*10^9^ cells/g. The bacterial assemblage in the freshly deployed wood (<1 day submerged) was dominated by sequences affiliated to *Gammaproteobacteria* (Figure 7). The most common genera indicated both a marine signature, with *Pseudoalteromonas* (strains of which have been shown to produce cellulase) and *Moritella* among the five most common genera, but also a terrestrial signature with typical plant-associated genera like *Burkholderia* (*Betaproteobacteria*), *Erwinia* (*Gammaproteobacteria)* and *Ralstonia* (*Betaproteobacteria*) (Table S5). In wood submerged for one year, *Alphaproteobacteria*, *Flavobacteria*, *Actinobacteria*, *Clostridia*, and *Bacteroidetes* showed higher relative sequence abundances when compared with the fresh wood (Figure 7, Table S4). These groups comprise several cellulolytic taxa: currently, the highest proportion of isolated cellulolytic bacteria are found within the *Firmicutes* (which include *Clostridia*) and *Actinobacteria* [92]. One conspicuous and highly sequence-abundant OTU_0.03_ affiliated with *Firmicutes* (*Clostridia*) was present in all wood and wood-chip sediment samples, indicating its potential role in wood utilization. *Clostridia* are obligate anaerobes and may indicate the presence of anoxic conditions in parts of the wood logs and chips that have developed due to wood degradation. The most common genus in wood submerged for one year was *Demequina* (*Actinobacteria*), for which an isolate (*Demequina aestuarii*) was shown to be closely related to *Cellulomonas fermentans* [95], allowing speculation that *Demequina* might play a significant role in wood degradation in the deep sea. Other observations of distinct genera included the recovery of sequences affiliated with *Teredinibacter* (see Xylophaga section), which were also found in clone libraries of one other wood-fall study [15], as well as sequences affiliated with the genera *Cellulophaga* (*Flavobacteria*) [96], [97] and *Phycisphaera* (*Planctomycetes*) [98]. Both genera have been previously isolated from marine algae, and were found in high relative abundances in the submerged wood, but rarely in the reference wood and sediments, indicating that they may also play a role in the degradation of woody material in the deep sea.

*Bacteroidetes* and *Firmicutes* had higher relative sequence abundances at and in the wood, whereas relative sequence abundances of *Acidobacteria* and *Actinobacteria* were highest in reference sediments away from wood experiments (Figure S2 and S3). *Bacteroidetes* may be responsible for the breakdown of a major fraction of complex organic matter in oceanic environments [99], [100], and both *Bacteroidetes* and *Firmicutes* have also been recovered from other wood falls [15] as well as from whale-fall influenced sediments [101]. *Deltaproteobacteria* were among the most common taxa both at the wood experiments as well as at the oxygen-limited site away from wood#1, consistent with findings at other wood falls [15]. *Deltaproteobacteria* included sulfate reducing members of the families *Desulfobacteraceae* and *Desulfobulbacea* (Table S7, S8, S9), which were also previously shown to be abundant at a whale fall and cold seeps [101]–[103]. A novel species of sulfate reducing bacteria (*Desulfovibrio piezophilus* sp. nov) has recently been isolated from other wood experiments in the Nile deep-ea fan [104], suggesting that several types of sulfate reducers may contribute to the degradation of cellulose at wood falls in the deep sea. *Acidobacteria* and *Actinobacteria*, in contrast, are typical representatives of deep-sea sediments and *Acidobacteria* are known to prefer oligotrophic conditions [105]–[107] which is consistent with their presence in undisturbed reference sediments. The relative importance of *Epsilonproteobacteria* at the oxygen-limited site #1 close to carbonate crusts (Table S3), is consistent with their dominance at seep and vent ecosystems [108]–[110]. Though *Epsilonproteobacteria* have also been found in association with whale falls [101], we could not confirm their relative importance at wood falls.

Despite the fact that differences in overall community structure were observed between the different wood experiments (Figure 6), our results suggest the presence of a core bacterial community in wood experiments submerged for 1 year, represented by a majority of sequences (18% shared OTU_0.03_ representing 72% of sequences) that clearly differed from the control wood and from background sediments (10 m away from wood#5). These bacterial types, some of which have been highlighted above (see also Table S3, S4, S5, S6, S7, S8, S9), may play an important role in the utilization of wood in the deep sea. However, metabolically active members of the community and the specific functions they perform need to be further investigated in the future. It also remains unknown, whether the majority of bacterial colonization takes place from the sediments or from the water column. Since on average 10% of OTU_0.03_ were shared between wood experiments and control wood#6, and 9% between wood experiments and their surrounding sediments, there must be additional sources of colonization, and future experiments should include sampling of the water column. In conclusion, we can confirm the hypotheses that sunken woods select for core communities of cellulose-degrading microorganisms – including sulfate reducing bacteria – which facilitate the development of sulfidic niches, building stepping stones for chemosynthetic life at these allochthonous habitats in the deep sea. Further studies are required to decipher the factors determining the temporal succession of bacterial communities at wood falls in the deep sea, and to identify the most abundant functional groups in enzymatic cellulose degradation, fermentation and reduction of sulfate to sulfide, supporting chemosynthetic life.

## Supporting Information

Figure S1 **Fine wood chips and fecal matter produced by wood-boring bivalves which had accumulated in a several centimeter thick layer around the wood experiments.** Scale is in millimeters.
(PDF)

Figure S2 **Bacterial community composition of the wood, wood-chip sediment boundary layer (at wood), and background sediment samples (away wood) of wood experiment#1 at the phylum and class level based on 454 massively parallel tag sequencing.**
(PDF)

Figure S3 **Bacterial community composition of the wood, wood-chip sediment boundary layer (at wood), and background sediment samples (away wood) of wood experiment#5 at the phylum and class level based on 454 massively parallel tag sequencing.**
(PDF)

Figure S4 **Shared proportions of OTU_0.03_ between wood experiments wood#1, wood#2, wood#5 (submerged for 1 year) and control wood#6 (submerged <1** **day).**
(PDF)

Table S1 Samples and measurements investigated in this study, with corresponding PANGAEA database references (PANGAEA® – Data Publisher for Earth & Environmental Science, doi:10.1594/PANGAEA). AODC: Acridine Orange Direct Cell Counts, ARISA: Automated Ribosomal Intergenic Spacer Analysis, 454 MPTS: 454 Massively Parallel Tag Sequencing.
(DOCX)

Table S2 Analysis of similarity (ANOSIM), testing for significant differences in bacterial community structures between the wood experiments as well as sediments around the wood experiments. *p<0.05, **p<0.01, ***p<0.001 after Bonferroni correction; (*) only significant without Bonferroni correction.
(DOCX)

Table S3 Cumulative list of the thirty most sequence abundant OTU_0.03_ for wood and sediment samples in alphabetical order. Colors indicate OTU_0.03_ unique to its sample origin. Sample names refer to control wood#6 that was submerged for less than 1 day, wood experiments that were submerged for 1 year, the wood-chip sediment boundary layers at wood experiments #1 and #5, and background sediments obtained 10 m away from wood experiments.
(DOC)

Table S4 Thirty most sequence abundant OTU_0.03_ common to the three wood experiments (wood#1, wood#2, wood#5) submerged for one year in alphabetical order.
(DOC)

Table S5 Thirty most sequence abundant OTU_0.03_ for control wood#6 submerged for less than one day in alphabetical order.
(DOC)

Table S6 Thirty most sequence abundant OTU_0.03_ for pelagic background sediments (10 m away from wood#5) in alphabetical order.
(DOC)

Table S7 Thirty most sequence abundant OTU_0.03_ for the wood-chip sediment boundary layer at wood#1 in alphabetical order.
(DOC)

Table S8 Thirty most sequence abundant OTU_0.03_ for the wood-chip sediment boundary layer at wood#5 in alphabetical order.
(DOC)

Table S9 Thirty most sequence abundant OTU_0.03_ for background sediments 10 m away from wood#1 in alphabetical order.
(DOC)
