# Supplementary material for: How Deep-Sea Wood Falls Sustain Chemosynthetic Life
Source: PLoS One. 2013 Jan 2;8(1):e53590. doi: 10.1371/journal.pone.0053590 (PMC3534711; doi:10.1371/journal.pone.0053590)
Supplement: Figure S1 — Fine wood chips and fecal matter produced by wood-boring bivalves which had accumulated in a several centimeter thick layer around the wood experiments. Scale is in millimeters. (PDF) [file pone.0053590.s001.pdf]

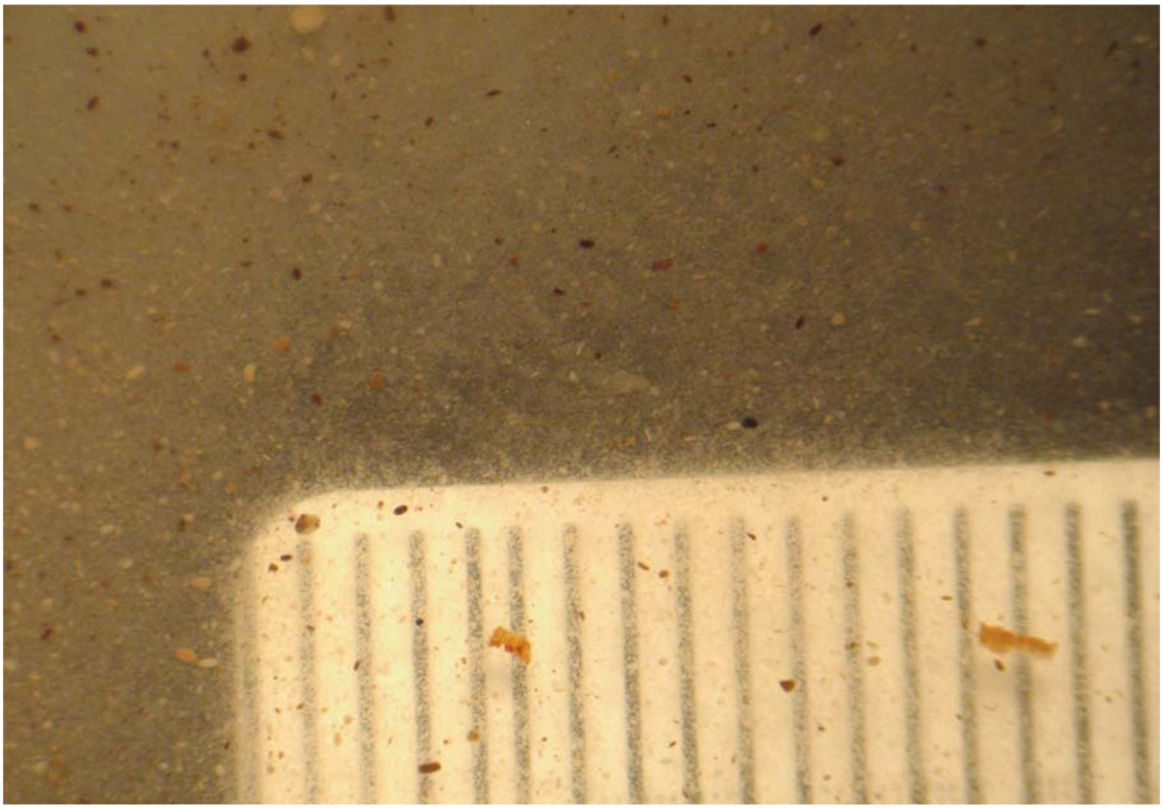

Figure S1 Fine wood chips and fecal matter produced by wood-boring bivalves which had accumulated in a several centimeter thick layer around the wood experiments. Scale is in millimeters.
