# Supplementary material for: How Deep-Sea Wood Falls Sustain Chemosynthetic Life
Source: PLoS One. 2013 Jan 2;8(1):e53590. doi: 10.1371/journal.pone.0053590 (PMC3534711; doi:10.1371/journal.pone.0053590)
Supplement: Figure S2 — Bacterial community composition of the wood, wood-chip sediment boundary layer (at wood), and background sediment samples (away wood) of wood experiment#1 at the phylum and class level based on 454 massively parallel tag sequencing. (PDF) [file pone.0053590.s002.pdf]

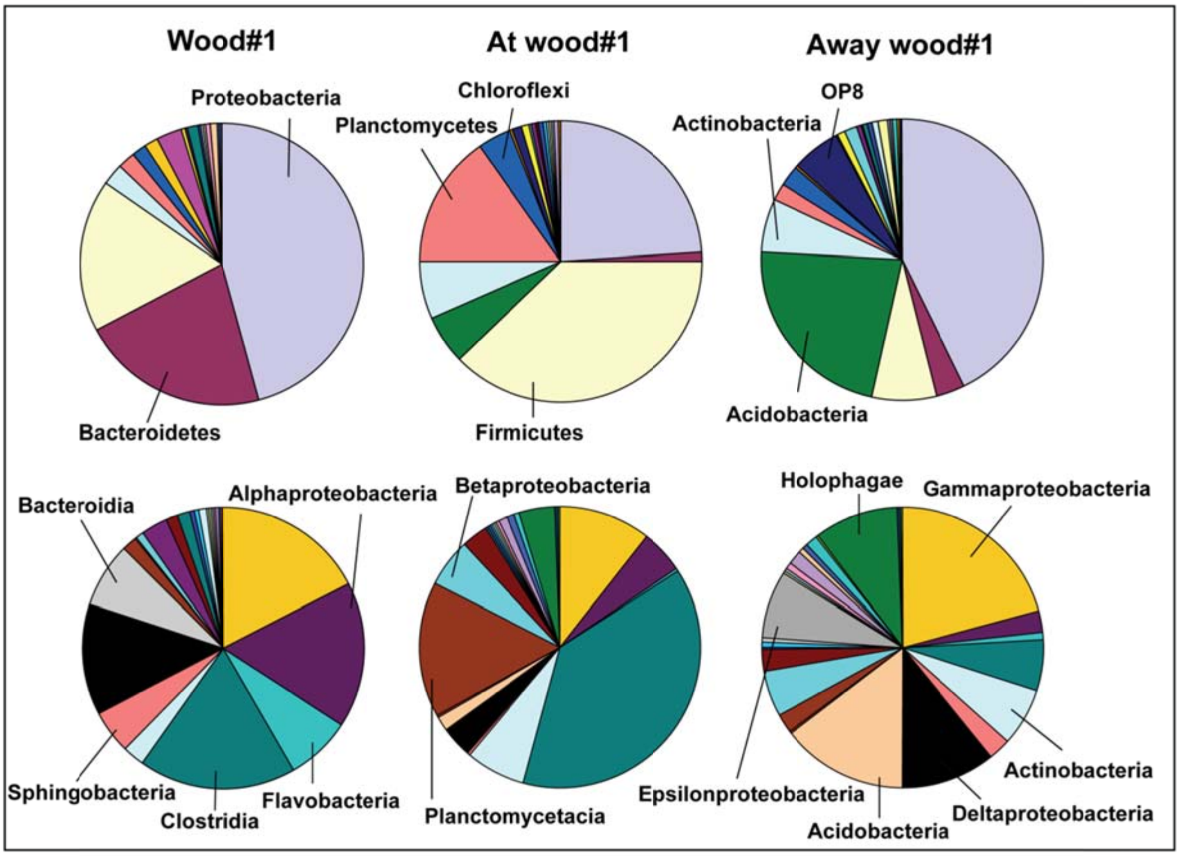

Figure S2 Bacterial community composition of the wood, wood-chip sediment boundary layer (at wood), and background sediment samples (away wood) of wood experiment#1 at the phylum and class level based on 454 massively parallel tag sequencing.
