# Supplementary material for: How Deep-Sea Wood Falls Sustain Chemosynthetic Life
Source: PLoS One. 2013 Jan 2;8(1):e53590. doi: 10.1371/journal.pone.0053590 (PMC3534711; doi:10.1371/journal.pone.0053590)
Supplement: Figure S3 — Bacterial community composition of the wood, wood-chip sediment boundary layer (at wood), and background sediment samples (away wood) of wood experiment#5 at the phylum and class level based on 454 massively parallel tag sequencing. (PDF) [file pone.0053590.s003.pdf]

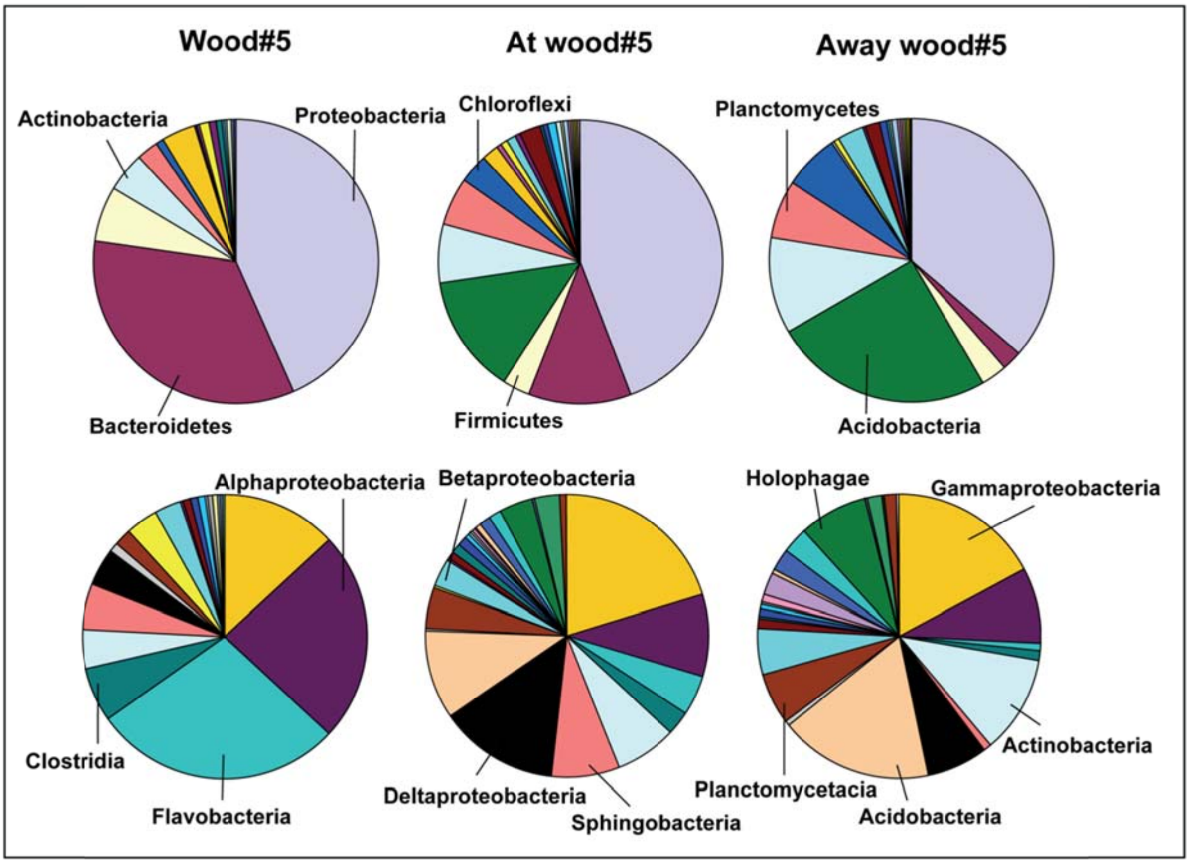

Figure S3 Bacterial community composition of the wood, wood-chip sediment boundary layer (at wood), and background sediment samples (away wood) of wood experiment#5 at the phylum and class level based on 454 massively parallel tag sequencing.
