# Supplementary material for: How Deep-Sea Wood Falls Sustain Chemosynthetic Life
Source: PLoS One. 2013 Jan 2;8(1):e53590. doi: 10.1371/journal.pone.0053590 (PMC3534711; doi:10.1371/journal.pone.0053590)
Supplement: Figure S4 — Shared proportions of OTU0.03 between wood experiments wood#1, wood#2, wood#5 (submerged for 1 year) and control wood#6 (submerged <1 day). (PDF) [file pone.0053590.s004.pdf]

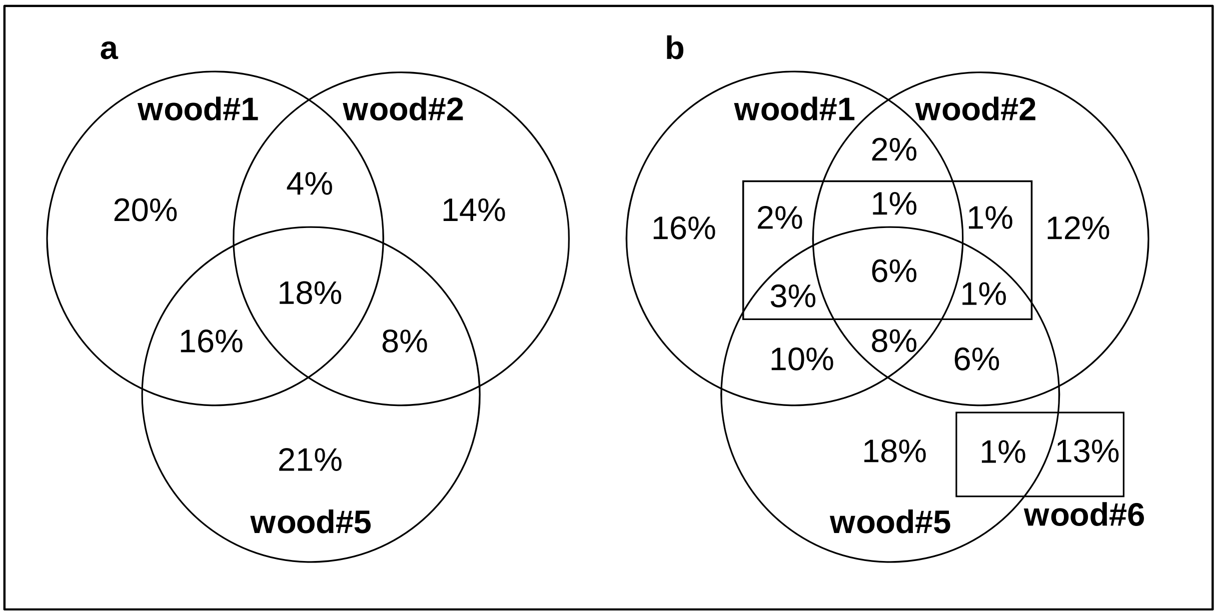

Figure S4 Shared proportions of $OTU_{0.03}$ between wood experiments wood#1, wood#2, wood#5 (submerged for 1 year) and control wood#6 (submerged <1 day).
