# Supplementary material for: How Deep-Sea Wood Falls Sustain Chemosynthetic Life
Source: PLoS One. 2013 Jan 2;8(1):e53590. doi: 10.1371/journal.pone.0053590 (PMC3534711; doi:10.1371/journal.pone.0053590)
Supplement: Table S1 — Samples and measurements investigated in this study, with corresponding PANGAEA database references (PANGAEA® – Data Publisher for Earth & Environmental Science, doi:10.1594/PANGAEA). AODC: Acridine Orange Direct Cell Counts, ARISA: Automated Ribosomal Intergenic Spacer Analysis, 454 MPTS: 454 Massively Parallel Tag Sequencing. (DOCX) [file pone.0053590.s005.docx]

**Table S1** Samples and measurements investigated in this study, with corresponding PANGAEA database references (PANGAEA® - Data Publisher for Earth & Environmental Science, doi:10.1594/PANGAEA). AODC: Acridine Orange Direct Cell Counts, ARISA: Automated Ribosomal Intergenic Spacer Analysis, 454 MPTS: 454 Massively Parallel Tag Sequencing.

| **Sample site** | **Measurement** | **PANGAEA database event label** |
| --- | --- | --- |
| Wood#1 (0.5 m) | AODC | MEDECO2-D338-PC-7 |
| Wood#1 (10 m) | AODC | MEDECO2-D338-PC-5 |
| Wood#5 (0.5 m) | AODC | MEDECO2-D339-PC-17 |
| Wood#5 (10 m) | AODC | MEDECO2-D339-PC-10 |
| Wood#1 (0.5 m) | DNA extraction  (ARISA, 454 MPTS) | MEDECO2-D338-PC-7, MEDECO2-D338-PC-8 |
| Wood#1 (10 m) | DNA extraction  (ARISA, 454 MPTS) | MEDECO2-D338-PC-6 |
| Wood#5 (0.5 m) | DNA extraction  (ARISA, 454 MPTS) | MEDECO2-D339-PC-16 |
| Wood#5 (10 m) | DNA extraction  (ARISA, 454 MPTS) | MEDECO2-D339-PC-10 |
| Wood#1 (0.5 m) | Benthic Chamber | MEDECO2-D338-CHAMB-4 |
| Wood#1 (10 m) | Benthic Chamber | MEDECO2-D338-CHAMB-5 |
| Wood#1 (0.5 m) | Ex situ microprofiler | MEDECO2-D338-PC-1 |
| Wood#1 (10 m) | Ex situ microprofiler | MEDECO2-D338-PC-5 |
| Wood#5 (0.5 m) | Ex situ microprofiler | MEDECO2-D338-PC-17 |
| Wood#5 (10 m) | Ex situ microprofiler | MEDECO2-D338-PC-10 |
| Wood#1 (0.5 m) | Sulfate reduction rate | MEDECO2-D338-PC-2 / -PC-7 / -PC-8 |
| Wood#1 (10 m) | Sulfate reduction rate | MEDECO2-D338-PC-9 / -PC-10 / -PC-12 |
| Wood#5 (0.5 m) | Sulfate reduction rate | MEDECO2-D338-PC-7 / -PC-8 / -PC-15 |
| Wood#1 (0.5 m) | Anaerobic oxidation of methane rate | MEDECO2-D339-PC-1, MEDECO2-D339-PC-3, MEDECO2-D339-PC-4 |
| Wood#1 (10 m) | Anaerobic oxidation of methane rate | MEDECO2-D338-PC-9, MEDECO2-D338-PC-10, MEDECO2-D338-PC-12 |
| Wood#5 (0.5 m) | Anaerobic oxidation of methane rate | MEDECO2-D339-PC-16, MEDECO2-D339-PC-17 |
| Wood#1 (0.5 m) | Pore water | MEDECO2-D338-PC-1, MEDECO2-D338-PC-3, MEDECO2-D338-PC-4, MEDECO2-D338-PC-7, MEDECO2-D338-PC-8 |
| Wood#1 (10 m) | Pore water | MEDECO2-D338-PC-5, MEDECO2-D338-PC-6, MEDECO2-D338-PC-9, MEDECO2-D338-PC-10, MEDECO2-D338-PC-11, MEDECO2-D338-PC-12 |
| Wood#5 (0.5 m) | Pore water | MEDECO2-D339-PC-7, MEDECO2-D338-PC-8, MEDECO2-D338-PC-16, MEDECO2-D338-PC-17 |
| Wood#5 (10 m) | Pore water | MEDECO2-D338-PC-9, MEDECO2-D338-PC-10 |
