# Supplementary material for: How Deep-Sea Wood Falls Sustain Chemosynthetic Life
Source: PLoS One. 2013 Jan 2;8(1):e53590. doi: 10.1371/journal.pone.0053590 (PMC3534711; doi:10.1371/journal.pone.0053590)
Supplement: Table S2 — Analysis of similarity (ANOSIM), testing for significant differences in bacterial community structures between the wood experiments as well as sediments around the wood experiments. *p<0.05, **p<0.01, ***p<0.001 after Bonferroni correction; (*) only significant without Bonferroni correction. (DOCX) [file pone.0053590.s006.docx]

**Table S2** Analysis of similarity (ANOSIM), testing for significant differences in bacterial community structures between the wood experiments as well as sediments around the wood experiments. *p<0.05, **p<0.01, ***p<0.001 after Bonferroni correction; (*) only significant without Bonferroni correction.

|  | **Wood#1** | **Wood#2** | **Wood#5** | **Wood#6** | **At wood#1** | **Away wood#1** | **At wood#5** |
| --- | --- | --- | --- | --- | --- | --- | --- |
| **Wood#1** |  |  |  |  |  |  |  |
| **Wood#2** | 0.48*** |  |  |  |  |  |  |
| **Wood#5** | 0.2 *** | 0.47*** |  |  |  |  |  |
| **Wood#6** | 1*** | 1*** | 1*** |  |  |  |  |
| **At wood#1** | 0.94** | 0.98** | 0.90** | 0.98(*) |  |  |  |
| **Away wood#1** | 0.99** | 0.99*** | 0.94** | 1(*) | 0.63 |  |  |
| **At wood#5** | 0.96*** | 0.97** | 0.85** | 0.81(*) | 0.21 | 0.42 |  |
| **Away wood#5** | 1** | 1** | 1** | 1(*) | 0.79 (*) | 0.52 (*) | 0.27 |
