# Supplementary material for: How Deep-Sea Wood Falls Sustain Chemosynthetic Life
Source: PLoS One. 2013 Jan 2;8(1):e53590. doi: 10.1371/journal.pone.0053590 (PMC3534711; doi:10.1371/journal.pone.0053590)
Supplement: Table S3 — Cumulative list of the thirty most sequence abundant OTU0.03 for wood and sediment samples in alphabetical order. Colors indicate OTU0.03 unique to its sample origin. Sample names refer to control wood#6 that was submerged for less than 1 day, wood experiments that were submerged for 1 year, the wood-chip sediment boundary layers at wood experiments #1 and #5, and background sediments obtained 10 m away from wood experiments. (DOC) [file pone.0053590.s007.doc]

**Table S3** Cumulative list of the thirty most sequence abundant OTU_0.03_ for wood and sediment samples in alphabetical order. Colors indicate OTU_0.03_ unique to its sample origin. Sample names refer to control wood#6 that was submerged for less than 1 day, wood experiments that were submerged for 1 year, the wood-chip sediment boundary layers at wood experiments #1 and #5, and background sediments obtained 10 m away from wood experiments.

| **OTU ID** | **Wood 1 day** | **Wood 1 year** | **at wood#1** | **at wood#5** | **away wood#5** | **away wood#1** | **Taxonomy** |
| --- | --- | --- | --- | --- | --- | --- | --- |
| Acidobacteria_03_135 |  |  |  |  |  | x | Bacteria;Acidobacteria;Acidobacteria;Acidobacteriales;Acidobacteriaceae |
| Acidobacteria_03_155 |  |  |  |  |  | x | Bacteria;Acidobacteria;Holophagae |
| Acidobacteria_03_178 |  |  |  |  |  | x | Bacteria;Acidobacteria;Holophagae |
| Acidobacteria_03_196 |  |  |  |  |  | x | Bacteria;Acidobacteria;Holophagae |
| Acidobacteria_03_20 |  |  |  | x | x |  | Bacteria;Acidobacteria;Acidobacteria;Acidobacteriales;Acidobacteriaceae |
| Acidobacteria_03_2233 | x |  |  |  |  |  | Bacteria;Acidobacteria;Acidobacteria;Acidobacteriales;Acidobacteriaceae;Terriglobus |
| Acidobacteria_03_271 |  |  |  |  |  | x | Bacteria;Acidobacteria;Holophagae |
| Acidobacteria_03_282 |  |  |  |  |  | x | Bacteria;Acidobacteria;Acidobacteria_Gp26;Unassigned;Unassigned;Gp26 |
| Acidobacteria_03_3 |  |  |  | x | x |  | Bacteria;Acidobacteria;Acidobacteria;Acidobacteriales;Acidobacteriaceae |
| Acidobacteria_03_31 |  |  |  | x | x |  | Bacteria;Acidobacteria;Acidobacteria;Acidobacteriales;Acidobacteriaceae |
| Acidobacteria_03_359 |  |  | x |  |  |  | Bacteria;Acidobacteria;Holophagae |
| Acidobacteria_03_39 |  |  |  |  |  | x | Bacteria;Acidobacteria;Acidobacteria_Gp26;Unassigned;Unassigned;Gp26 |
| Acidobacteria_03_43 |  |  |  | x | x | x | Bacteria;Acidobacteria;Acidobacteria;Acidobacteriales;Acidobacteriaceae |
| Acidobacteria_03_455 |  |  | x |  |  |  | Bacteria;Acidobacteria;Holophagae |
| Acidobacteria_03_500 |  |  |  |  | x |  | Bacteria;Acidobacteria;Acidobacteria;Acidobacteriales;Acidobacteriaceae |
| Acidobacteria_03_71 |  |  |  |  |  | x | Bacteria;Acidobacteria;Holophagae |
| Acidobacteria_03_812 |  |  | x |  |  |  | Bacteria;Acidobacteria;Holophagae |
| Acidobacteria_03_862 |  |  |  |  | x |  | Bacteria;Acidobacteria;Holophagae |
| Actinobacteria_03_11 |  |  | x |  | x | x | Bacteria;Actinobacteria;Actinobacteria;Acidimicrobiales |
| Actinobacteria_03_113 |  | x |  |  |  |  | Bacteria;Actinobacteria;Actinobacteria;Acidimicrobiales;Iamiaceae;Iamia |
| Actinobacteria_03_184 |  | x |  |  |  |  | Bacteria;Actinobacteria;Actinobacteria;Actinomycetales;Unassigned;Demequina |
| Actinobacteria_03_185 |  |  |  |  | x |  | Bacteria;Actinobacteria;Actinobacteria;Acidimicrobiales |
| Actinobacteria_03_186 |  |  |  | x | x |  | Bacteria;Actinobacteria;Actinobacteria;Acidimicrobiales |
| Actinobacteria_03_290 | x |  |  |  |  |  | Bacteria;Actinobacteria;Actinobacteria;Actinomycetales;Microbacteriaceae |
| Actinobacteria_03_3 | x |  | x |  | x |  | Bacteria;Actinobacteria;Actinobacteria;Actinomycetales;Propionibacteriaceae;Propionibacterium |
| Actinobacteria_03_35 |  |  |  |  |  | x | Bacteria;Actinobacteria;Actinobacteria;Acidimicrobiales |
| Actinobacteria_03_52 |  |  | x |  |  | x | Bacteria;Actinobacteria;Actinobacteria;Coriobacteriales;Coriobacteriaceae;Gordonibacter |
| Actinobacteria_03_53 |  |  |  |  |  | x | Bacteria;Actinobacteria;Actinobacteria;Acidimicrobiales |
| Actinobacteria_03_663 |  |  | x |  |  |  | Bacteria;Actinobacteria;Actinobacteria |
| Actinobacteria_03_71 |  |  |  | x | x |  | Bacteria;Actinobacteria;Actinobacteria;Acidimicrobiales |
| Actinobacteria_03_76 |  | x |  |  |  |  | Bacteria;Actinobacteria;Actinobacteria;Actinomycetales;Unassigned;Demequina |
| Actinobacteria_03_825 |  |  | x |  |  |  | Bacteria;Actinobacteria;Actinobacteria;Acidimicrobiales |
| Alphaproteobacteria_03_119 |  | x |  |  |  |  | Bacteria;Proteobacteria;Alphaproteobacteria;Rhodospirillales;Rhodospirillaceae |
| Alphaproteobacteria_03_130 |  | x |  |  |  |  | Bacteria;Proteobacteria;Alphaproteobacteria;Rhodobacterales;Rhodobacteraceae;Tropicimonas |
| Alphaproteobacteria_03_139 |  | x |  |  |  |  | Bacteria;Proteobacteria;Alphaproteobacteria;Rhodobacterales;Rhodobacteraceae |
| Alphaproteobacteria_03_150 |  |  | x |  | x |  | Bacteria;Proteobacteria;Alphaproteobacteria;Rhizobiales;Methylobacteriaceae;Methylobacterium |
| Alphaproteobacteria_03_260 |  | x |  | x |  |  | Bacteria;Proteobacteria;Alphaproteobacteria;Rhodobacterales;Rhodobacteraceae |
| Alphaproteobacteria_03_28 | x |  | x |  |  |  | Bacteria;Proteobacteria;Alphaproteobacteria;Sphingomonadales;Sphingomonadaceae;Sphingomonas |
| Alphaproteobacteria_03_29 |  | x |  | x |  |  | Bacteria;Proteobacteria;Alphaproteobacteria;Rhodobacterales;Rhodobacteraceae |
| Alphaproteobacteria_03_3 | x |  |  |  |  |  | Bacteria;Proteobacteria;Alphaproteobacteria;Sphingomonadales;Sphingomonadaceae;Sphingomonas |
| Alphaproteobacteria_03_337 | x |  |  |  |  |  | Bacteria;Proteobacteria;Alphaproteobacteria;Rhizobiales;Methylocystaceae |
| Alphaproteobacteria_03_349 |  | x |  |  |  |  | Bacteria;Proteobacteria;Alphaproteobacteria;Rhodobacterales;Rhodobacteraceae;Thalassobacter |
| Alphaproteobacteria_03_367 |  | x |  |  |  |  | Bacteria;Proteobacteria;Alphaproteobacteria;Rhodobacterales;Rhodobacteraceae;Roseovarius |
| Alphaproteobacteria_03_37 | x |  |  |  |  |  | Bacteria;Proteobacteria;Alphaproteobacteria;Sphingomonadales;Sphingomonadaceae |
| Alphaproteobacteria_03_419 |  | x |  |  |  |  | Bacteria;Proteobacteria;Alphaproteobacteria;Rhodobacterales;Rhodobacteraceae |
| Alphaproteobacteria_03_437 |  | x |  |  |  |  | Bacteria;Proteobacteria;Alphaproteobacteria;Rhodobacterales;Rhodobacteraceae |
| Alphaproteobacteria_03_452 |  |  |  |  | x |  | Bacteria;Proteobacteria;Alphaproteobacteria;Rhodospirillales;Rhodospirillaceae;Pelagibius |
| Alphaproteobacteria_03_569 |  | x |  |  |  |  | Bacteria;Proteobacteria;Alphaproteobacteria;Caulobacterales;Hyphomonadaceae;Hirschia;baltica |
| Alphaproteobacteria_03_59 |  |  | x | x | x |  | Bacteria;Proteobacteria;Alphaproteobacteria;Rhizobiales;Methylobacteriaceae;Methylobacterium |
| Alphaproteobacteria_03_663 |  | x |  |  |  |  | Bacteria;Proteobacteria;Alphaproteobacteria;Rhizobiales;Phyllobacteriaceae |
| BacteriaNA_03_110 |  |  | x |  |  |  | Bacteria |
| Bacteroidetes_03_1117 |  |  |  | x |  |  | Bacteria;Bacteroidetes;Sphingobacteria;Sphingobacteriales;Flammeovirgaceae;Reichenbachiella |
| Bacteroidetes_03_12 |  | x |  |  |  |  | Bacteria;Bacteroidetes;Flavobacteria;Flavobacteriales;Flavobacteriaceae |
| Bacteroidetes_03_149 |  |  |  |  |  | x | Bacteria;Bacteroidetes;Sphingobacteria;Sphingobacteriales;Flammeovirgaceae;Reichenbachiella |
| Bacteroidetes_03_194 |  | x |  |  |  |  | Bacteria;Bacteroidetes;Flavobacteria;Flavobacteriales;Flavobacteriaceae |
| Bacteroidetes_03_23 | x | x |  |  |  |  | Bacteria;Bacteroidetes;Flavobacteria;Flavobacteriales;Flavobacteriaceae |
| Bacteroidetes_03_317 |  | x |  |  |  |  | Bacteria;Bacteroidetes;Flavobacteria;Flavobacteriales;Flavobacteriaceae;Polaribacter |
| Bacteroidetes_03_356 |  | x |  |  |  |  | Bacteria;Bacteroidetes;Sphingobacteria;Sphingobacteriales;Chitinophagaceae |
| Bacteroidetes_03_389 |  | x |  |  |  |  | Bacteria;Bacteroidetes;Bacteroidia;Bacteroidales;Marinilabiaceae |
| Bacteroidetes_03_429 |  | x |  | x |  |  | Bacteria;Bacteroidetes;Flavobacteria;Flavobacteriales;Flavobacteriaceae |
| Bacteroidetes_03_6 |  | x |  | x |  |  | Bacteria;Bacteroidetes;Flavobacteria;Flavobacteriales;Flavobacteriaceae |
| Bacteroidetes_03_612 | x |  |  |  |  |  | Bacteria;Bacteroidetes;Sphingobacteria;Sphingobacteriales;Unassigned;Prolixibacter |
| Betaproteobacteria_03_1 | x |  | x | x | x | x | Bacteria;Proteobacteria;Betaproteobacteria;Burkholderiales;Burkholderiaceae;Ralstonia |
| Betaproteobacteria_03_152 |  | x |  |  |  |  | Bacteria;Proteobacteria;Betaproteobacteria;Neisseriales;Neisseriaceae;Conchiformibius;steedae |
| Betaproteobacteria_03_19 |  |  | x |  |  |  | Bacteria;Proteobacteria;Betaproteobacteria;Burkholderiales;Comamonadaceae |
| Betaproteobacteria_03_446 | x |  |  |  |  |  | Bacteria;Proteobacteria;Betaproteobacteria;Burkholderiales;Burkholderiaceae;Burkholderia;andropogonis |
| Betaproteobacteria_03_519 | x |  |  |  |  |  | Bacteria;Proteobacteria;Betaproteobacteria;Burkholderiales;Burkholderiaceae;Burkholderia |
| Betaproteobacteria_03_804 | x |  |  |  |  |  | Bacteria;Proteobacteria;Betaproteobacteria;Burkholderiales;Burkholderiaceae;Burkholderia |
| Chloroflexi_03_11 |  |  | x |  |  |  | Bacteria;Chloroflexi;Anaerolineae;Anaerolineales;Anaerolinaceae |
| Chloroflexi_03_24 |  |  | x |  |  | x | Bacteria;Chloroflexi;Anaerolineae;Anaerolineales;Anaerolinaceae |
| Chloroflexi_03_35 |  |  |  |  | x |  | Bacteria;Chloroflexi;Caldilineae;Caldilineales |
| Chloroflexi_03_38 |  |  |  |  |  | x | Bacteria;Chloroflexi;Anaerolineae;Anaerolineales;Anaerolinaceae |
| Chloroflexi_03_92 |  |  | x |  |  |  | Bacteria;Chloroflexi;Caldilineae;Caldilineales |
| Cyanobacteria_03_28 |  |  | x |  |  |  | Bacteria;Cyanobacteria |
| Deltaproteobacteria_03_106 |  | x |  |  |  |  | Bacteria;Proteobacteria;Deltaproteobacteria;Desulfobacterales;Desulfobulbaceae;Desulforhopalus |
| Deltaproteobacteria_03_24 |  |  |  |  |  | x | Bacteria;Proteobacteria;Deltaproteobacteria;Desulfobacterales;Desulfobulbaceae;Desulfocapsa |
| Deltaproteobacteria_03_27 |  |  |  | x |  |  | Bacteria;Proteobacteria;Deltaproteobacteria;Desulfobacterales;Desulfobacteraceae;Desulfobacula |
| Deltaproteobacteria_03_283 |  |  |  | x |  |  | Bacteria;Proteobacteria;Deltaproteobacteria;Desulfobacterales;Desulfobacteraceae;Desulfobacula |
| Deltaproteobacteria_03_341 |  |  |  |  | x |  | Bacteria;Proteobacteria;Deltaproteobacteria |
| Deltaproteobacteria_03_421 |  |  |  | x |  |  | Bacteria;Proteobacteria;Deltaproteobacteria;Desulfobacterales;Desulfobacteraceae;Desulfotignum |
| Deltaproteobacteria_03_50 |  |  |  |  |  | x | Bacteria;Proteobacteria;Deltaproteobacteria;Desulfobacterales;Desulfobulbaceae;Desulfobulbus |
| Deltaproteobacteria_03_55 |  |  | x |  |  |  | Bacteria;Proteobacteria;Deltaproteobacteria;Desulfobacterales;Desulfobulbaceae |
| Deltaproteobacteria_03_737 |  |  |  | x |  |  | Bacteria;Proteobacteria;Deltaproteobacteria;Desulfobacterales;Desulfobulbaceae;Desulfopila |
| Deltaproteobacteria_03_977 |  |  |  | x |  |  | Bacteria;Proteobacteria;Deltaproteobacteria;Myxococcales |
| Epsilonproteobacteria_03_19 |  |  |  |  |  | x | Bacteria;Proteobacteria;Epsilonproteobacteria;Campylobacterales;Helicobacteraceae;Sulfurovum |
| Epsilonproteobacteria_03_26 |  |  |  |  |  | x | Bacteria;Proteobacteria;Epsilonproteobacteria;Campylobacterales;Helicobacteraceae;Sulfurovum |
| Epsilonproteobacteria_03_58 |  |  |  |  |  | x | Bacteria;Proteobacteria;Epsilonproteobacteria;Campylobacterales;Helicobacteraceae;Sulfurovum |
| Firmicutes_03_116 | x | x | x | x |  |  | Bacteria;Firmicutes;Clostridia;Clostridiales;Lachnospiraceae |
| Firmicutes_03_1456 |  |  |  |  |  | x | Bacteria;Firmicutes;Bacilli;Lactobacillales;Streptococcaceae;Streptococcus |
| Firmicutes_03_562 |  | x |  |  |  |  | Bacteria;Firmicutes;Clostridia;Clostridiales;Lachnospiraceae |
| Firmicutes_03_627 |  |  |  |  |  | x | Bacteria;Firmicutes;Clostridia;Clostridiales;Ruminococcaceae;Oscillibacter |
| Firmicutes_03_692 |  | x |  |  |  |  | Bacteria;Firmicutes;Clostridia;Clostridiales;Peptostreptococcaceae;Fusibacter |
| Gammaproteobacteria_03_1 |  |  | x |  |  |  | Bacteria;Proteobacteria;Gammaproteobacteria;Enterobacteriales;Enterobacteriaceae |
| Gammaproteobacteria_03_10 |  |  |  |  | x | x | Bacteria;Proteobacteria;Gammaproteobacteria |
| Gammaproteobacteria_03_1134 |  |  |  |  |  | x | Bacteria;Proteobacteria;Gammaproteobacteria |
| Gammaproteobacteria_03_12 | x |  |  |  |  |  | Bacteria;Proteobacteria;Gammaproteobacteria |
| Gammaproteobacteria_03_126 | x |  |  |  |  |  | Bacteria;Proteobacteria;Gammaproteobacteria;Alteromonadales;Pseudoalteromonadaceae;Pseudoalteromonas |
| Gammaproteobacteria_03_13 | x |  |  |  |  |  | Bacteria;Proteobacteria;Gammaproteobacteria |
| Gammaproteobacteria_03_151 |  |  |  |  | x |  | Bacteria;Proteobacteria;Gammaproteobacteria |
| Gammaproteobacteria_03_197 |  |  |  |  | x |  | Bacteria;Proteobacteria;Gammaproteobacteria;Alteromonadales;Alteromonadaceae |
| Gammaproteobacteria_03_2 |  |  | x |  |  |  | Bacteria;Proteobacteria;Gammaproteobacteria;Enterobacteriales;Enterobacteriaceae |
| Gammaproteobacteria_03_211 | x |  |  |  |  |  | Bacteria;Proteobacteria;Gammaproteobacteria;Alteromonadales;Moritellaceae;Moritella |
| Gammaproteobacteria_03_23 | x |  |  |  |  |  | Bacteria;Proteobacteria;Gammaproteobacteria;Pseudomonadales;Pseudomonadaceae;Pseudomonas |
| Gammaproteobacteria_03_242 |  |  |  | x |  |  | Bacteria;Proteobacteria;Gammaproteobacteria |
| Gammaproteobacteria_03_244 |  |  |  | x |  |  | Bacteria;Proteobacteria;Gammaproteobacteria;Alteromonadales;Alteromonadaceae;Haliea |
| Gammaproteobacteria_03_245 | x |  |  |  |  |  | Bacteria;Proteobacteria;Gammaproteobacteria;Pasteurellales;Pasteurellaceae |
| Gammaproteobacteria_03_249 | x |  |  |  |  |  | Bacteria;Proteobacteria;Gammaproteobacteria;Enterobacteriales;Enterobacteriaceae;Erwinia |
| Gammaproteobacteria_03_254 | x |  |  |  |  |  | Bacteria;Proteobacteria;Gammaproteobacteria;Alteromonadales;Pseudoalteromonadaceae;Pseudoalteromonas |
| Gammaproteobacteria_03_270 |  |  |  |  | x |  | Bacteria;Proteobacteria;Gammaproteobacteria |
| Gammaproteobacteria_03_3 |  |  | x |  |  |  | Bacteria;Proteobacteria;Gammaproteobacteria;Pseudomonadales;Moraxellaceae;Acinetobacter |
| Gammaproteobacteria_03_300 | x |  |  |  |  |  | Bacteria;Proteobacteria;Gammaproteobacteria;Vibrionales;Vibrionaceae;Vibrio |
| Gammaproteobacteria_03_339 | x |  |  |  |  |  | Bacteria;Proteobacteria;Gammaproteobacteria;Xanthomonadales;Xanthomonadaceae |
| Gammaproteobacteria_03_36 |  |  |  |  | x |  | Bacteria;Proteobacteria;Gammaproteobacteria;Xanthomonadales;Sinobacteraceae |
| Gammaproteobacteria_03_394 | x |  |  |  |  |  | Bacteria;Proteobacteria;Gammaproteobacteria;Vibrionales;Vibrionaceae;Vibrio |
| Gammaproteobacteria_03_4 | x |  | x |  |  |  | Bacteria;Proteobacteria;Gammaproteobacteria;Pseudomonadales;Pseudomonadaceae;Pseudomonas |
| Gammaproteobacteria_03_424 | x |  |  |  |  |  | Bacteria;Proteobacteria;Gammaproteobacteria;Alteromonadales;Pseudoalteromonadaceae;Pseudoalteromonas |
| Gammaproteobacteria_03_435 |  | x |  |  |  |  | Bacteria;Proteobacteria;Gammaproteobacteria;Thiotrichales;Thiotrichaceae |
| Gammaproteobacteria_03_463 |  |  | x | x | x | x | Bacteria;Proteobacteria;Gammaproteobacteria;Legionellales;Coxiellaceae;Coxiella |
| Gammaproteobacteria_03_477 |  |  | x | x | x |  | Bacteria;Proteobacteria;Gammaproteobacteria;Legionellales;Coxiellaceae;Coxiella |
| Gammaproteobacteria_03_53 |  |  |  |  |  | x | Bacteria;Proteobacteria;Gammaproteobacteria;Acidithiobacillales;Acidithiobacillaceae;Acidithiobacillus |
| Gammaproteobacteria_03_536 |  | x |  |  |  |  | Bacteria;Proteobacteria;Gammaproteobacteria |
| Gammaproteobacteria_03_56 | x |  |  |  |  |  | Bacteria;Proteobacteria;Gammaproteobacteria;Alteromonadales;Pseudoalteromonadaceae;Pseudoalteromonas |
| Gammaproteobacteria_03_595 |  |  | x |  |  |  | Bacteria;Proteobacteria;Gammaproteobacteria;Legionellales;Coxiellaceae;Coxiella |
| Gammaproteobacteria_03_632 |  |  |  |  | x |  | Bacteria;Proteobacteria;Gammaproteobacteria |
| Gammaproteobacteria_03_651 |  |  |  |  | x |  | Bacteria;Proteobacteria;Gammaproteobacteria;Oceanospirillales;Oceanospirillaceae;Marinomonas |
| Gammaproteobacteria_03_70 |  | x |  |  |  |  | Bacteria;Proteobacteria;Gammaproteobacteria;Vibrionales;Vibrionaceae;Vibrio |
| Gammaproteobacteria_03_71 |  |  |  | x | x |  | Bacteria;Proteobacteria;Gammaproteobacteria;Xanthomonadales;Sinobacteraceae |
| Gammaproteobacteria_03_753 | x |  |  |  |  |  | Bacteria;Proteobacteria;Gammaproteobacteria |
| Gammaproteobacteria_03_8 | x |  |  |  |  |  | Bacteria;Proteobacteria;Gammaproteobacteria;Alteromonadales;Pseudoalteromonadaceae;Pseudoalteromonas |
| Gammaproteobacteria_03_854 |  |  |  | x |  |  | Bacteria;Proteobacteria;Gammaproteobacteria;Alteromonadales;Colwelliaceae;Colwellia |
| Gemmatimonadetes_03_1 |  |  |  |  | x |  | Bacteria;Gemmatimonadetes;Gemmatimonadetes |
| Gemmatimonadetes_03_10 |  |  |  |  |  | x | Bacteria;Gemmatimonadetes;Gemmatimonadetes |
| Nitrospirae_03_72 |  |  |  | x |  |  | Bacteria;Nitrospirae;Nitrospira;Nitrospirales;Nitrospiraceae |
| OP8_03_104 |  |  | x |  |  |  | Bacteria;OP8 |
| OP8_03_29 |  |  |  |  |  | x | Bacteria;OP8 |
| Planctomycetes_03_1 |  |  | x | x | x |  | Bacteria;Planctomycetes;Planctomycetacia;Planctomycetales;Planctomycetaceae |
| Planctomycetes_03_12 |  |  |  | x |  |  | Bacteria;Planctomycetes;Planctomycetacia;Planctomycetales;Planctomycetaceae;Blastopirellula |
| Planctomycetes_03_3 |  |  | x | x | x |  | Bacteria;Planctomycetes;Planctomycetacia;Planctomycetales;Planctomycetaceae |
| Verrucomicrobia_03_37 |  | x |  |  |  |  | Bacteria;Verrucomicrobia;Verrucomicrobiae;Verrucomicrobiales;Verrucomicrobiaceae;Haloferula |
