# Supplementary material for: How Deep-Sea Wood Falls Sustain Chemosynthetic Life
Source: PLoS One. 2013 Jan 2;8(1):e53590. doi: 10.1371/journal.pone.0053590 (PMC3534711; doi:10.1371/journal.pone.0053590)
Supplement: Table S4 — Thirty most sequence abundant OTU0.03 common to the three wood experiments (wood#1, wood#2, wood#5) submerged for one year in alphabetical order. (DOC) [file pone.0053590.s008.doc]

**Table S4** Thirty most sequence abundant OTU_0.03_ common to the three wood experiments (wood#1, wood#2, wood#5) submerged for one year in alphabetical order.

| **OTU ID** | **Sequence abundance** | **Relative sequence abundance** | **Taxonomy** |
| --- | --- | --- | --- |
| Actinobacteria_03_113 | 1001 | 1.21E-02 | Bacteria;Actinobacteria;Actinobacteria;Acidimicrobiales;Iamiaceae;Iamia |
| Actinobacteria_03_184 | 829 | 1.01E-02 | Bacteria;Actinobacteria;Actinobacteria;Actinomycetales;Unassigned;Demequina |
| Actinobacteria_03_76 | 2459 | 3.00E-02 | Bacteria;Actinobacteria;Actinobacteria;Actinomycetales;Unassigned;Demequina |
| Alphaproteobacteria_03_119 | 669 | 8.16E-03 | Bacteria;Proteobacteria;Alphaproteobacteria;Rhodospirillales;Rhodospirillaceae |
| Alphaproteobacteria_03_130 | 606 | 7.39E-03 | Bacteria;Proteobacteria;Alphaproteobacteria;Rhodobacterales;Rhodobacteraceae;Tropicimonas |
| Alphaproteobacteria_03_139 | 523 | 6.38E-03 | Bacteria;Proteobacteria;Alphaproteobacteria;Rhodobacterales;Rhodobacteraceae |
| Alphaproteobacteria_03_260 | 640 | 7.81E-03 | Bacteria;Proteobacteria;Alphaproteobacteria;Rhodobacterales;Rhodobacteraceae |
| Alphaproteobacteria_03_29 | 1445 | 1.76E-02 | Bacteria;Proteobacteria;Alphaproteobacteria;Rhodobacterales;Rhodobacteraceae |
| Alphaproteobacteria_03_349 | 576 | 7.03E-03 | Bacteria;Proteobacteria;Alphaproteobacteria;Rhodobacterales;Rhodobacteraceae;Thalassobacter |
| Alphaproteobacteria_03_367 | 572 | 6.98E-03 | Bacteria;Proteobacteria;Alphaproteobacteria;Rhodobacterales;Rhodobacteraceae;Roseovarius |
| Alphaproteobacteria_03_419 | 596 | 7.27E-03 | Bacteria;Proteobacteria;Alphaproteobacteria;Rhodobacterales;Rhodobacteraceae |
| Alphaproteobacteria_03_437 | 951 | 1.16E-02 | Bacteria;Proteobacteria;Alphaproteobacteria;Rhodobacterales;Rhodobacteraceae |
| Alphaproteobacteria_03_569 | 654 | 7.98E-03 | Bacteria;Proteobacteria;Alphaproteobacteria;Caulobacterales;Hyphomonadaceae;Hirschia;baltica |
| Alphaproteobacteria_03_663 | 551 | 6.72E-03 | Bacteria;Proteobacteria;Alphaproteobacteria;Rhizobiales;Phyllobacteriaceae |
| Bacteroidetes_03_12 | 1154 | 1.41E-02 | Bacteria;Bacteroidetes;Flavobacteria;Flavobacteriales;Flavobacteriaceae |
| Bacteroidetes_03_194 | 1378 | 1.68E-02 | Bacteria;Bacteroidetes;Flavobacteria;Flavobacteriales;Flavobacteriaceae |
| Bacteroidetes_03_23 | 8112 | 9.90E-02 | Bacteria;Bacteroidetes;Flavobacteria;Flavobacteriales;Flavobacteriaceae |
| Bacteroidetes_03_317 | 683 | 8.33E-03 | Bacteria;Bacteroidetes;Flavobacteria;Flavobacteriales;Flavobacteriaceae;Polaribacter |
| Bacteroidetes_03_356 | 972 | 1.19E-02 | Bacteria;Bacteroidetes;Sphingobacteria;Sphingobacteriales;Chitinophagaceae |
| Bacteroidetes_03_389 | 954 | 1.16E-02 | Bacteria;Bacteroidetes;Bacteroidia;Bacteroidales;Marinilabiaceae |
| Bacteroidetes_03_429 | 750 | 9.15E-03 | Bacteria;Bacteroidetes;Flavobacteria;Flavobacteriales;Flavobacteriaceae |
| Bacteroidetes_03_6 | 1815 | 2.21E-02 | Bacteria;Bacteroidetes;Flavobacteria;Flavobacteriales;Flavobacteriaceae |
| Betaproteobacteria_03_152 | 1436 | 1.75E-02 | Bacteria;Proteobacteria;Betaproteobacteria;Neisseriales;Neisseriaceae;Conchiformibius;steedae |
| Deltaproteobacteria_03_106 | 547 | 6.67E-03 | Bacteria;Proteobacteria;Deltaproteobacteria;Desulfobacterales;Desulfobulbaceae;Desulforhopalus |
| Firmicutes_03_116 | 2033 | 2.48E-02 | Bacteria;Firmicutes;Clostridia;Clostridiales;Lachnospiraceae |
| Firmicutes_03_562 | 698 | 8.51E-03 | Bacteria;Firmicutes;Clostridia;Clostridiales;Lachnospiraceae |
| Firmicutes_03_692 | 706 | 8.61E-03 | Bacteria;Firmicutes;Clostridia;Clostridiales;Peptostreptococcaceae;Fusibacter |
| Gammaproteobacteria_03_435 | 1232 | 1.50E-02 | Bacteria;Proteobacteria;Gammaproteobacteria;Thiotrichales;Thiotrichaceae |
| Gammaproteobacteria_03_536 | 764 | 9.32E-03 | Bacteria;Proteobacteria;Gammaproteobacteria |
| Verrucomicrobia_03_37 | 1016 | 1.24E-02 | Bacteria;Verrucomicrobia;Verrucomicrobiae;Verrucomicrobiales;Verrucomicrobiaceae;Haloferula |
