# Supplementary material for: How Deep-Sea Wood Falls Sustain Chemosynthetic Life
Source: PLoS One. 2013 Jan 2;8(1):e53590. doi: 10.1371/journal.pone.0053590 (PMC3534711; doi:10.1371/journal.pone.0053590)
Supplement: Table S5 — Thirty most sequence abundant OTU0.03 for control wood#6 submerged for less than one day in alphabetical order. (DOC) [file pone.0053590.s009.doc]

**Table S5** Thirty most sequence abundant OTU_0.03_ for control wood#6 submerged for less than one day in alphabetical order.

| **OTU ID** | **Sequence abundance** | **Relative sequence abundance** | **Taxonomy** |
| --- | --- | --- | --- |
| Acidobacteria_03_2233 | 20 | 1.66E-03 | Bacteria;Acidobacteria;Acidobacteria;Acidobacteriales;Acidobacteriaceae;Terriglobus |
| Actinobacteria_03_290 | 51 | 4.23E-03 | Bacteria;Actinobacteria;Actinobacteria;Actinomycetales;Microbacteriaceae |
| Actinobacteria_03_3 | 55 | 4.57E-03 | Bacteria;Actinobacteria;Actinobacteria;Actinomycetales;Propionibacteriaceae;Propionibacterium |
| Alphaproteobacteria_03_28 | 68 | 5.64E-03 | Bacteria;Proteobacteria;Alphaproteobacteria;Sphingomonadales;Sphingomonadaceae;Sphingomonas |
| Alphaproteobacteria_03_3 | 19 | 1.58E-03 | Bacteria;Proteobacteria;Alphaproteobacteria;Sphingomonadales;Sphingomonadaceae;Sphingomonas |
| Alphaproteobacteria_03_337 | 30 | 2.49E-03 | Bacteria;Proteobacteria;Alphaproteobacteria;Rhizobiales;Methylocystaceae |
| Alphaproteobacteria_03_37 | 19 | 1.58E-03 | Bacteria;Proteobacteria;Alphaproteobacteria;Sphingomonadales;Sphingomonadaceae |
| Bacteroidetes_03_23 | 62 | 5.15E-03 | Bacteria;Bacteroidetes;Flavobacteria;Flavobacteriales;Flavobacteriaceae |
| Betaproteobacteria_03_1 | 70 | 5.81E-03 | Bacteria;Proteobacteria;Betaproteobacteria;Burkholderiales;Burkholderiaceae;Ralstonia |
| Betaproteobacteria_03_446 | 43 | 3.57E-03 | Bacteria;Proteobacteria;Betaproteobacteria;Burkholderiales;Burkholderiaceae;Burkholderia;andropogonis |
| Betaproteobacteria_03_519 | 88 | 7.30E-03 | Bacteria;Proteobacteria;Betaproteobacteria;Burkholderiales;Burkholderiaceae;Burkholderia |
| Betaproteobacteria_03_804 | 25 | 2.08E-03 | Bacteria;Proteobacteria;Betaproteobacteria;Burkholderiales;Burkholderiaceae;Burkholderia |
| Firmicutes_03_116 | 48 | 3.98E-03 | Bacteria;Firmicutes;Clostridia;Clostridiales;Lachnospiraceae |
| Gammaproteobacteria_03_12 | 160 | 1.33E-02 | Bacteria;Proteobacteria;Gammaproteobacteria |
| Gammaproteobacteria_03_126 | 37 | 3.07E-03 | Bacteria;Proteobacteria;Gammaproteobacteria;Alteromonadales;Pseudoalteromonadaceae;Pseudoalteromonas |
| Gammaproteobacteria_03_13 | 92 | 7.64E-03 | Bacteria;Proteobacteria;Gammaproteobacteria |
| Gammaproteobacteria_03_211 | 1628 | 1.35E-01 | Bacteria;Proteobacteria;Gammaproteobacteria;Alteromonadales;Moritellaceae;Moritella |
| Gammaproteobacteria_03_23 | 68 | 5.64E-03 | Bacteria;Proteobacteria;Gammaproteobacteria;Pseudomonadales;Pseudomonadaceae;Pseudomonas |
| Gammaproteobacteria_03_245 | 80 | 6.64E-03 | Bacteria;Proteobacteria;Gammaproteobacteria;Pasteurellales;Pasteurellaceae |
| Gammaproteobacteria_03_249 | 86 | 7.14E-03 | Bacteria;Proteobacteria;Gammaproteobacteria;Enterobacteriales;Enterobacteriaceae;Erwinia |
| Gammaproteobacteria_03_254 | 159 | 1.32E-02 | Bacteria;Proteobacteria;Gammaproteobacteria;Alteromonadales;Pseudoalteromonadaceae;Pseudoalteromonas |
| Gammaproteobacteria_03_300 | 186 | 1.54E-02 | Bacteria;Proteobacteria;Gammaproteobacteria;Vibrionales;Vibrionaceae;Vibrio |
| Gammaproteobacteria_03_339 | 231 | 1.92E-02 | Bacteria;Proteobacteria;Gammaproteobacteria;Xanthomonadales;Xanthomonadaceae |
| Gammaproteobacteria_03_394 | 52 | 4.32E-03 | Bacteria;Proteobacteria;Gammaproteobacteria;Vibrionales;Vibrionaceae;Vibrio |
| Gammaproteobacteria_03_4 | 59 | 4.90E-03 | Bacteria;Proteobacteria;Gammaproteobacteria;Pseudomonadales;Pseudomonadaceae;Pseudomonas |
| Gammaproteobacteria_03_424 | 76 | 6.31E-03 | Bacteria;Proteobacteria;Gammaproteobacteria;Alteromonadales;Pseudoalteromonadaceae;Pseudoalteromonas |
| Gammaproteobacteria_03_56 | 625 | 5.19E-02 | Bacteria;Proteobacteria;Gammaproteobacteria;Alteromonadales;Pseudoalteromonadaceae;Pseudoalteromonas |
| Gammaproteobacteria_03_70 | 476 | 3.95E-02 | Bacteria;Proteobacteria;Gammaproteobacteria;Vibrionales;Vibrionaceae;Vibrio |
| Gammaproteobacteria_03_753 | 33 | 2.74E-03 | Bacteria;Proteobacteria;Gammaproteobacteria |
| Gammaproteobacteria_03_8 | 5752 | 4.77E-01 | Bacteria;Proteobacteria;Gammaproteobacteria;Alteromonadales;Pseudoalteromonadaceae;Pseudoalteromonas |
