# Supplementary material for: How Deep-Sea Wood Falls Sustain Chemosynthetic Life
Source: PLoS One. 2013 Jan 2;8(1):e53590. doi: 10.1371/journal.pone.0053590 (PMC3534711; doi:10.1371/journal.pone.0053590)
Supplement: Table S6 — Thirty most sequence abundant OTU0.03 for pelagic background sediments (10 m away from wood#5) in alphabetical order. (DOC) [file pone.0053590.s010.doc]

**Table S6** Thirty most sequence abundant OTU_0.03_ for pelagic background sediments (10 m away from wood#5) in alphabetical order.

| **OTU ID** | **Sequence abundance** | **Relative sequence abundance** | **Taxonomy** |
| --- | --- | --- | --- |
| Acidobacteria_03_20 | 138 | 1.16E-02 | Bacteria;Acidobacteria;Acidobacteria;Acidobacteriales;Acidobacteriaceae |
| Acidobacteria_03_3 | 117 | 9.85E-03 | Bacteria;Acidobacteria;Acidobacteria;Acidobacteriales;Acidobacteriaceae |
| Acidobacteria_03_31 | 82 | 6.90E-03 | Bacteria;Acidobacteria;Acidobacteria;Acidobacteriales;Acidobacteriaceae |
| Acidobacteria_03_43 | 549 | 4.62E-02 | Bacteria;Acidobacteria;Acidobacteria;Acidobacteriales;Acidobacteriaceae |
| Acidobacteria_03_500 | 73 | 6.14E-03 | Bacteria;Acidobacteria;Acidobacteria;Acidobacteriales;Acidobacteriaceae |
| Acidobacteria_03_862 | 110 | 9.26E-03 | Bacteria;Acidobacteria;Holophagae |
| Actinobacteria_03_11 | 66 | 5.55E-03 | Bacteria;Actinobacteria;Actinobacteria;Acidimicrobiales |
| Actinobacteria_03_185 | 99 | 8.33E-03 | Bacteria;Actinobacteria;Actinobacteria;Acidimicrobiales |
| Actinobacteria_03_186 | 147 | 1.24E-02 | Bacteria;Actinobacteria;Actinobacteria;Acidimicrobiales |
| Actinobacteria_03_3 | 195 | 1.64E-02 | Bacteria;Actinobacteria;Actinobacteria;Actinomycetales;Propionibacteriaceae;Propionibacterium |
| Actinobacteria_03_71 | 66 | 5.55E-03 | Bacteria;Actinobacteria;Actinobacteria;Acidimicrobiales |
| Alphaproteobacteria_03_150 | 101 | 8.50E-03 | Bacteria;Proteobacteria;Alphaproteobacteria;Rhizobiales;Methylobacteriaceae;Methylobacterium |
| Alphaproteobacteria_03_452 | 138 | 1.16E-02 | Bacteria;Proteobacteria;Alphaproteobacteria;Rhodospirillales;Rhodospirillaceae;Pelagibius |
| Alphaproteobacteria_03_59 | 66 | 5.55E-03 | Bacteria;Proteobacteria;Alphaproteobacteria;Rhizobiales;Methylobacteriaceae;Methylobacterium |
| Betaproteobacteria_03_1 | 416 | 3.50E-02 | Bacteria;Proteobacteria;Betaproteobacteria;Burkholderiales;Burkholderiaceae;Ralstonia |
| Chloroflexi_03_35 | 167 | 1.41E-02 | Bacteria;Chloroflexi;Caldilineae;Caldilineales |
| Deltaproteobacteria_03_341 | 75 | 6.31E-03 | Bacteria;Proteobacteria;Deltaproteobacteria |
| Gammaproteobacteria_03_10 | 64 | 5.39E-03 | Bacteria;Proteobacteria;Gammaproteobacteria |
| Gammaproteobacteria_03_151 | 62 | 5.22E-03 | Bacteria;Proteobacteria;Gammaproteobacteria |
| Gammaproteobacteria_03_197 | 66 | 5.55E-03 | Bacteria;Proteobacteria;Gammaproteobacteria;Alteromonadales;Alteromonadaceae |
| Gammaproteobacteria_03_270 | 68 | 5.72E-03 | Bacteria;Proteobacteria;Gammaproteobacteria |
| Gammaproteobacteria_03_36 | 98 | 8.25E-03 | Bacteria;Proteobacteria;Gammaproteobacteria;Xanthomonadales;Sinobacteraceae |
| Gammaproteobacteria_03_463 | 116 | 9.76E-03 | Bacteria;Proteobacteria;Gammaproteobacteria;Legionellales;Coxiellaceae;Coxiella |
| Gammaproteobacteria_03_477 | 58 | 4.88E-03 | Bacteria;Proteobacteria;Gammaproteobacteria;Legionellales;Coxiellaceae;Coxiella |
| Gammaproteobacteria_03_632 | 55 | 4.63E-03 | Bacteria;Proteobacteria;Gammaproteobacteria |
| Gammaproteobacteria_03_651 | 60 | 5.05E-03 | Bacteria;Proteobacteria;Gammaproteobacteria;Oceanospirillales;Oceanospirillaceae;Marinomonas |
| Gammaproteobacteria_03_71 | 219 | 1.84E-02 | Bacteria;Proteobacteria;Gammaproteobacteria;Xanthomonadales;Sinobacteraceae |
| Gemmatimonadetes_03_1 | 77 | 6.48E-03 | Bacteria;Gemmatimonadetes;Gemmatimonadetes |
| Planctomycetes_03_1 | 86 | 7.24E-03 | Bacteria;Planctomycetes;Planctomycetacia;Planctomycetales;Planctomycetaceae |
| Planctomycetes_03_3 | 206 | 1.73E-02 | Bacteria;Planctomycetes;Planctomycetacia;Planctomycetales;Planctomycetaceae |
