# Supplementary material for: How Deep-Sea Wood Falls Sustain Chemosynthetic Life
Source: PLoS One. 2013 Jan 2;8(1):e53590. doi: 10.1371/journal.pone.0053590 (PMC3534711; doi:10.1371/journal.pone.0053590)
Supplement: Table S7 — Thirty most sequence abundant OTU0.03 for the wood-chip sediment boundary layer at wood#1 in alphabetical order. (DOC) [file pone.0053590.s011.doc]

**Table S7** Thirty most sequence abundant OTU_0.03_ for the wood-chip sediment boundary layer at wood#1 in alphabetical order.

| **OTU ID** | **Sequence abundance** | **Relative sequence abundance** | **Taxonomy** |
| --- | --- | --- | --- |
| Acidobacteria_03_359 | 94 | 4.86E-03 | Bacteria;Acidobacteria;Holophagae |
| Acidobacteria_03_455 | 245 | 1.27E-02 | Bacteria;Acidobacteria;Holophagae |
| Acidobacteria_03_812 | 113 | 5.85E-03 | Bacteria;Acidobacteria;Holophagae |
| Actinobacteria_03_11 | 353 | 1.83E-02 | Bacteria;Actinobacteria;Actinobacteria;Acidimicrobiales |
| Actinobacteria_03_3 | 124 | 6.42E-03 | Bacteria;Actinobacteria;Actinobacteria;Actinomycetales;Propionibacteriaceae;Propionibacterium |
| Actinobacteria_03_52 | 81 | 4.19E-03 | Bacteria;Actinobacteria;Actinobacteria;Coriobacteriales;Coriobacteriaceae;Gordonibacter |
| Actinobacteria_03_663 | 134 | 6.93E-03 | Bacteria;Actinobacteria;Actinobacteria |
| Actinobacteria_03_825 | 87 | 4.50E-03 | Bacteria;Actinobacteria;Actinobacteria;Acidimicrobiales |
| Alphaproteobacteria_03_150 | 229 | 1.18E-02 | Bacteria;Proteobacteria;Alphaproteobacteria;Rhizobiales;Methylobacteriaceae;Methylobacterium |
| Alphaproteobacteria_03_28 | 115 | 5.95E-03 | Bacteria;Proteobacteria;Alphaproteobacteria;Sphingomonadales;Sphingomonadaceae;Sphingomonas |
| Alphaproteobacteria_03_59 | 154 | 7.97E-03 | Bacteria;Proteobacteria;Alphaproteobacteria;Rhizobiales;Methylobacteriaceae;Methylobacterium |
| BacteriaNA_03_110 | 75 | 3.88E-03 | Bacteria |
| Betaproteobacteria_03_1 | 608 | 3.15E-02 | Bacteria;Proteobacteria;Betaproteobacteria;Burkholderiales;Burkholderiaceae;Ralstonia |
| Betaproteobacteria_03_19 | 106 | 5.48E-03 | Bacteria;Proteobacteria;Betaproteobacteria;Burkholderiales;Comamonadaceae |
| Chloroflexi_03_11 | 224 | 1.16E-02 | Bacteria;Chloroflexi;Anaerolineae;Anaerolineales;Anaerolinaceae |
| Chloroflexi_03_24 | 121 | 6.26E-03 | Bacteria;Chloroflexi;Anaerolineae;Anaerolineales;Anaerolinaceae |
| Chloroflexi_03_92 | 62 | 3.21E-03 | Bacteria;Chloroflexi;Caldilineae;Caldilineales |
| Cyanobacteria_03_28 | 67 | 3.47E-03 | Bacteria;Cyanobacteria |
| Deltaproteobacteria_03_55 | 302 | 1.56E-02 | Bacteria;Proteobacteria;Deltaproteobacteria;Desulfobacterales;Desulfobulbaceae |
| Firmicutes_03_116 | 6885 | 3.56E-01 | Bacteria;Firmicutes;Clostridia;Clostridiales;Lachnospiraceae |
| Gammaproteobacteria_03_1 | 80 | 4.14E-03 | Bacteria;Proteobacteria;Gammaproteobacteria;Enterobacteriales;Enterobacteriaceae |
| Gammaproteobacteria_03_2 | 68 | 3.52E-03 | Bacteria;Proteobacteria;Gammaproteobacteria;Enterobacteriales;Enterobacteriaceae |
| Gammaproteobacteria_03_3 | 168 | 8.69E-03 | Bacteria;Proteobacteria;Gammaproteobacteria;Pseudomonadales;Moraxellaceae;Acinetobacter |
| Gammaproteobacteria_03_4 | 72 | 3.72E-03 | Bacteria;Proteobacteria;Gammaproteobacteria;Pseudomonadales;Pseudomonadaceae;Pseudomonas |
| Gammaproteobacteria_03_463 | 104 | 5.38E-03 | Bacteria;Proteobacteria;Gammaproteobacteria;Legionellales;Coxiellaceae;Coxiella |
| Gammaproteobacteria_03_477 | 227 | 1.17E-02 | Bacteria;Proteobacteria;Gammaproteobacteria;Legionellales;Coxiellaceae;Coxiella |
| Gammaproteobacteria_03_595 | 179 | 9.26E-03 | Bacteria;Proteobacteria;Gammaproteobacteria;Legionellales;Coxiellaceae;Coxiella |
| OP8_03_104 | 70 | 3.62E-03 | Bacteria;OP8 |
| Planctomycetes_03_1 | 458 | 2.37E-02 | Bacteria;Planctomycetes;Planctomycetacia;Planctomycetales;Planctomycetaceae |
| Planctomycetes_03_3 | 2314 | 1.20E-01 | Bacteria;Planctomycetes;Planctomycetacia;Planctomycetales;Planctomycetaceae |
