# Supplementary material for: How Deep-Sea Wood Falls Sustain Chemosynthetic Life
Source: PLoS One. 2013 Jan 2;8(1):e53590. doi: 10.1371/journal.pone.0053590 (PMC3534711; doi:10.1371/journal.pone.0053590)
Supplement: Table S8 — Thirty most sequence abundant OTU0.03 for the wood-chip sediment boundary layer at wood#5 in alphabetical order. (DOC) [file pone.0053590.s012.doc]

**Table S8** Thirty most sequence abundant OTU_0.03_ for the wood-chip sediment boundary layer at wood#5 in alphabetical order.

| **OTU ID** | **Sequence abundance** | **Relative sequence abundance** | **Taxonomy** |
| --- | --- | --- | --- |
| Acidobacteria_03_20 | 72 | 8.19E-03 | Bacteria;Acidobacteria;Acidobacteria;Acidobacteriales;Acidobacteriaceae |
| Acidobacteria_03_3 | 108 | 1.23E-02 | Bacteria;Acidobacteria;Acidobacteria;Acidobacteriales;Acidobacteriaceae |
| Acidobacteria_03_31 | 68 | 7.73E-03 | Bacteria;Acidobacteria;Acidobacteria;Acidobacteriales;Acidobacteriaceae |
| Acidobacteria_03_43 | 140 | 1.59E-02 | Bacteria;Acidobacteria;Acidobacteria;Acidobacteriales;Acidobacteriaceae |
| Actinobacteria_03_186 | 91 | 1.03E-02 | Bacteria;Actinobacteria;Actinobacteria;Acidimicrobiales |
| Actinobacteria_03_71 | 51 | 5.80E-03 | Bacteria;Actinobacteria;Actinobacteria;Acidimicrobiales |
| Alphaproteobacteria_03_260 | 50 | 5.69E-03 | Bacteria;Proteobacteria;Alphaproteobacteria;Rhodobacterales;Rhodobacteraceae |
| Alphaproteobacteria_03_29 | 68 | 7.73E-03 | Bacteria;Proteobacteria;Alphaproteobacteria;Rhodobacterales;Rhodobacteraceae |
| Alphaproteobacteria_03_59 | 53 | 6.03E-03 | Bacteria;Proteobacteria;Alphaproteobacteria;Rhizobiales;Methylobacteriaceae;Methylobacterium |
| Bacteroidetes_03_1117 | 80 | 9.10E-03 | Bacteria;Bacteroidetes;Sphingobacteria;Sphingobacteriales;Flammeovirgaceae;Reichenbachiella |
| Bacteroidetes_03_429 | 65 | 7.39E-03 | Bacteria;Bacteroidetes;Flavobacteria;Flavobacteriales;Flavobacteriaceae |
| Bacteroidetes_03_6 | 148 | 1.68E-02 | Bacteria;Bacteroidetes;Flavobacteria;Flavobacteriales;Flavobacteriaceae |
| Bacteroidetes_03_612 | 136 | 1.55E-02 | Bacteria;Bacteroidetes;Sphingobacteria;Sphingobacteriales;Unassigned;Prolixibacter |
| Betaproteobacteria_03_1 | 121 | 1.38E-02 | Bacteria;Proteobacteria;Betaproteobacteria;Burkholderiales;Burkholderiaceae;Ralstonia |
| Deltaproteobacteria_03_27 | 79 | 8.98E-03 | Bacteria;Proteobacteria;Deltaproteobacteria;Desulfobacterales;Desulfobacteraceae;Desulfobacula |
| Deltaproteobacteria_03_283 | 76 | 8.64E-03 | Bacteria;Proteobacteria;Deltaproteobacteria;Desulfobacterales;Desulfobacteraceae;Desulfobacula |
| Deltaproteobacteria_03_421 | 44 | 5.00E-03 | Bacteria;Proteobacteria;Deltaproteobacteria;Desulfobacterales;Desulfobacteraceae;Desulfotignum |
| Deltaproteobacteria_03_737 | 66 | 7.51E-03 | Bacteria;Proteobacteria;Deltaproteobacteria;Desulfobacterales;Desulfobulbaceae;Desulfopila |
| Deltaproteobacteria_03_977 | 75 | 8.53E-03 | Bacteria;Proteobacteria;Deltaproteobacteria;Myxococcales |
| Firmicutes_03_116 | 156 | 1.77E-02 | Bacteria;Firmicutes;Clostridia;Clostridiales;Lachnospiraceae |
| Gammaproteobacteria_03_242 | 67 | 7.62E-03 | Bacteria;Proteobacteria;Gammaproteobacteria |
| Gammaproteobacteria_03_244 | 77 | 8.76E-03 | Bacteria;Proteobacteria;Gammaproteobacteria;Alteromonadales;Alteromonadaceae;Haliea |
| Gammaproteobacteria_03_463 | 44 | 5.00E-03 | Bacteria;Proteobacteria;Gammaproteobacteria;Legionellales;Coxiellaceae;Coxiella |
| Gammaproteobacteria_03_477 | 56 | 6.37E-03 | Bacteria;Proteobacteria;Gammaproteobacteria;Legionellales;Coxiellaceae;Coxiella |
| Gammaproteobacteria_03_71 | 45 | 5.12E-03 | Bacteria;Proteobacteria;Gammaproteobacteria;Xanthomonadales;Sinobacteraceae |
| Gammaproteobacteria_03_854 | 168 | 1.91E-02 | Bacteria;Proteobacteria;Gammaproteobacteria;Alteromonadales;Colwelliaceae;Colwellia |
| Nitrospirae_03_72 | 123 | 1.40E-02 | Bacteria;Nitrospirae;Nitrospira;Nitrospirales;Nitrospiraceae |
| Planctomycetes_03_1 | 47 | 5.35E-03 | Bacteria;Planctomycetes;Planctomycetacia;Planctomycetales;Planctomycetaceae |
| Planctomycetes_03_12 | 54 | 6.14E-03 | Bacteria;Planctomycetes;Planctomycetacia;Planctomycetales;Planctomycetaceae;Blastopirellula |
| Planctomycetes_03_3 | 65 | 7.39E-03 | Bacteria;Planctomycetes;Planctomycetacia;Planctomycetales;Planctomycetaceae |
