# Supplementary material for: How Deep-Sea Wood Falls Sustain Chemosynthetic Life
Source: PLoS One. 2013 Jan 2;8(1):e53590. doi: 10.1371/journal.pone.0053590 (PMC3534711; doi:10.1371/journal.pone.0053590)
Supplement: Table S9 — Thirty most sequence abundant OTU0.03 for background sediments 10 m away from wood#1 in alphabetical order. (DOC) [file pone.0053590.s013.doc]

**Table S9** Thirty most sequence abundant OTU_0.03_ for background sediments 10 m away from wood#1 in alphabetical order.

| **OTU ID** | **Sequence abundance** | **Relative sequence abundance** | **Taxonomy** |
| --- | --- | --- | --- |
| Acidobacteria_03_135 | 103 | 7.70E-03 | Bacteria;Acidobacteria;Acidobacteria;Acidobacteriales;Acidobacteriaceae |
| Acidobacteria_03_155 | 343 | 2.57E-02 | Bacteria;Acidobacteria;Holophagae |
| Acidobacteria_03_178 | 74 | 5.53E-03 | Bacteria;Acidobacteria;Holophagae |
| Acidobacteria_03_196 | 155 | 1.16E-02 | Bacteria;Acidobacteria;Holophagae |
| Acidobacteria_03_271 | 109 | 8.15E-03 | Bacteria;Acidobacteria;Holophagae |
| Acidobacteria_03_282 | 70 | 5.24E-03 | Bacteria;Acidobacteria;Acidobacteria_Gp26;Unassigned;Unassigned;Gp26 |
| Acidobacteria_03_39 | 1180 | 8.83E-02 | Bacteria;Acidobacteria;Acidobacteria_Gp26;Unassigned;Unassigned;Gp26 |
| Acidobacteria_03_43 | 80 | 5.98E-03 | Bacteria;Acidobacteria;Acidobacteria;Acidobacteriales;Acidobacteriaceae |
| Acidobacteria_03_71 | 88 | 6.58E-03 | Bacteria;Acidobacteria;Holophagae |
| Actinobacteria_03_11 | 91 | 6.81E-03 | Bacteria;Actinobacteria;Actinobacteria;Acidimicrobiales |
| Actinobacteria_03_35 | 141 | 1.05E-02 | Bacteria;Actinobacteria;Actinobacteria;Acidimicrobiales |
| Actinobacteria_03_52 | 103 | 7.70E-03 | Bacteria;Actinobacteria;Actinobacteria;Coriobacteriales;Coriobacteriaceae;Gordonibacter |
| Actinobacteria_03_53 | 70 | 5.24E-03 | Bacteria;Actinobacteria;Actinobacteria;Acidimicrobiales |
| Bacteroidetes_03_149 | 176 | 1.32E-02 | Bacteria;Bacteroidetes;Sphingobacteria;Sphingobacteriales;Flammeovirgaceae;Reichenbachiella |
| Betaproteobacteria_03_1 | 517 | 3.87E-02 | Bacteria;Proteobacteria;Betaproteobacteria;Burkholderiales;Burkholderiaceae;Ralstonia |
| Chloroflexi_03_24 | 73 | 5.46E-03 | Bacteria;Chloroflexi;Anaerolineae;Anaerolineales;Anaerolinaceae |
| Chloroflexi_03_38 | 78 | 5.83E-03 | Bacteria;Chloroflexi;Anaerolineae;Anaerolineales;Anaerolinaceae |
| Deltaproteobacteria_03_24 | 128 | 9.57E-03 | Bacteria;Proteobacteria;Deltaproteobacteria;Desulfobacterales;Desulfobulbaceae;Desulfocapsa |
| Deltaproteobacteria_03_50 | 284 | 2.12E-02 | Bacteria;Proteobacteria;Deltaproteobacteria;Desulfobacterales;Desulfobulbaceae;Desulfobulbus |
| Epsilonproteobacteria_03_19 | 431 | 3.22E-02 | Bacteria;Proteobacteria;Epsilonproteobacteria;Campylobacterales;Helicobacteraceae;Sulfurovum |
| Epsilonproteobacteria_03_26 | 121 | 9.05E-03 | Bacteria;Proteobacteria;Epsilonproteobacteria;Campylobacterales;Helicobacteraceae;Sulfurovum |
| Epsilonproteobacteria_03_58 | 187 | 1.40E-02 | Bacteria;Proteobacteria;Epsilonproteobacteria;Campylobacterales;Helicobacteraceae;Sulfurovum |
| Firmicutes_03_1456 | 122 | 9.12E-03 | Bacteria;Firmicutes;Bacilli;Lactobacillales;Streptococcaceae;Streptococcus |
| Firmicutes_03_627 | 624 | 4.67E-02 | Bacteria;Firmicutes;Clostridia;Clostridiales;Ruminococcaceae;Oscillibacter |
| Gammaproteobacteria_03_10 | 84 | 6.28E-03 | Bacteria;Proteobacteria;Gammaproteobacteria |
| Gammaproteobacteria_03_1134 | 146 | 1.09E-02 | Bacteria;Proteobacteria;Gammaproteobacteria;Legionellales;Coxiellaceae |
| Gammaproteobacteria_03_463 | 103 | 7.70E-03 | Bacteria;Proteobacteria;Gammaproteobacteria;Legionellales;Coxiellaceae;Coxiella |
| Gammaproteobacteria_03_53 | 111 | 8.30E-03 | Bacteria;Proteobacteria;Gammaproteobacteria;Acidithiobacillales;Acidithiobacillaceae;Acidithiobacillus |
| Gemmatimonadetes_03_10 | 90 | 6.73E-03 | Bacteria;Gemmatimonadetes;Gemmatimonadetes |
| OP8_03_29 | 436 | 3.26E-02 | Bacteria;OP8 |
